# Single‐cell transcriptomes reveal heterogeneity of high‐grade serous ovarian carcinoma

**DOI:** 10.1002/ctm2.500

**Published:** 2021-08-04

**Authors:** Qian Hao, Jiajia Li, Qinghua Zhang, Fei Xu, Bangxiang Xie, Hua Lu, Xiaohua Wu, Xiang Zhou

**Affiliations:** ^1^ Fudan University Shanghai Cancer Center and Institutes of Biomedical Sciences Fudan University Shanghai China; ^2^ Department of Gynecologic Oncology, Fudan University Shanghai Cancer Center Fudan University Shanghai China; ^3^ Department of Oncology, Shanghai Medical College Fudan University Shanghai China; ^4^ Beijing YouAn Hospital, Capital Medical University Beijing Institute of Hepatology Beijing China; ^5^ Department of Biochemistry & Molecular Biology and Tulane Cancer Center Tulane University School of Medicine New Orleans Louisiana; ^6^ Key Laboratory of Breast Cancer in Shanghai, Fudan University Shanghai Cancer Center Fudan University Shanghai China; ^7^ Shanghai Key Laboratory of Medical Epigenetics, International Co‐laboratory of Medical Epigenetics and Metabolism, Ministry of Science and Technology, Institutes of Biomedical Sciences Fudan University Shanghai China

**Keywords:** chemoresistance, gene regulatory network, intercellular communication, metastasis, single‐cell RNA‐seq

## Abstract

**Background:**

High‐grade serous ovarian carcinoma (HGSOC) is the most common and aggressive histotype of epithelial ovarian cancer. The heterogeneity and molecular basis of this disease remain incompletely understood.

**Methods:**

To address this question, we have performed a single‐cell transcriptomics analysis of matched primary and metastatic HGSOC samples.

**Results:**

A total of 13 571 cells are categorized into six distinct cell types, including epithelial cells, fibroblast cells, T cells, B cells, macrophages, and endothelial cells. A subset of aggressive epithelial cells with hyperproliferative and drug‐resistant potentials is identified. Several new markers that are highly expressed in epithelial cells are characterized, and their roles in ovarian cancer cell growth and migration are further confirmed. Dysregulation of multiple signaling pathways, including the translational machinery, is associated with ovarian cancer metastasis through the trajectory analysis. Moreover, single‐cell regulatory network inference and clustering (SCENIC) analysis reveals the gene regulatory networks and suggests the JUN signaling pathway as a potential therapeutic target for treatment of ovarian cancer, which is validated using the JUN/AP‐1 inhibitor T‐5224. Finally, our study depicts the epithelial‐fibroblast cell communication atlas and identifies several important receptor‐ligand complexes in ovarian cancer development.

**Conclusions:**

This study uncovers new molecular features and the potential therapeutic target of HGSOC, which would advance the understanding and treatment of the disease.

## Graphical abstract

(1) Single‐cell transcriptomics analysis of HGSOC samples reveals six distinct cell types.

(2) Characterization of epithelial cell subclusters identifies novel markers for HGSOC.

(3) Subclustering fibroblast cells suggests their functional heterogeneity.

(4) Our study also uncovers the JUN pathway and several ligand‐receptor signals as potential drivers for HGSOC.

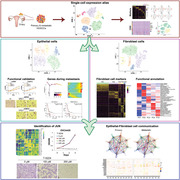

## 1 INTRODUCTION

High‐grade serous ovarian carcinoma (HGSOC) is the most aggressive gynecological malignancy and one of the leading causes of cancer death in women worldwide. Our knowledge about the molecular etiology and clinical pathology of HGSOC is greatly advanced. Nevertheless, the overall prognosis of the disease has been barely improved for decades.^1^ Primary debulking surgery followed by chemotherapy has become the frontline treatment for patients with HGSOC since the 1980s. However, most patients die from the relapsed disease, because treatment resistance eventually emerges in 80‐90% of cases initially diagnosed with the late stage and metastatic spread.^2^ Thus, thoroughly elucidating the mechanisms underlying ovarian cancer metastasis and refractoriness remains an active area of investigation, which would be of significant benefit to the development of therapeutic approaches and survival of patients.

Mutation of the tumor suppressor gene *TP53* is both an early event and an invariant feature of HGSOC that is characterized by extreme chromosomal instability.^3^, ^4^ While wild‐type p53 protein is regarded as “a guardian of the genome” by inducing cell cycle arrest and DNA repair,^5^, ^6^ p53 mutation not only abrogates the wild‐type activity of p53, but also endows mutant p53 with oncogenic function, namely “gain of function,” to promote metastasis and chemoresistance of ovarian cancer.^7^, ^8^, ^9^ Besides *TP53* gene mutation, deficiency of the homologous recombination (HR) DNA repair pathway is found in approximately 50% of HGSOCs,^3^, ^4^ which arises mainly from mutations in *BRCA1* and *BRCA2* ^10^ and, in some cases, from other components of the HR pathway.^11^ The common amplifications encoding *CCNE1*, *MYC*, and *MECOM* are present in more than 20% of tumors, which empowers cancer progression and provides potential therapeutic targets.^3^, ^4^ In addition, gene expression profiling identified four molecular subtypes of HGSOC, termed “immunoreactive,” “differentiated,” “proliferative,” and “mesenchymal,”^3^, ^12^ although this stratification has not been integrated into the clinical setting. However, bulk tumors were analyzed and characterized in the above studies, while HGSOCs display intratumoral heterogeneity that profoundly undermines our knowledge about the pathogenesis and influences the therapeutic outcomes of the disease.

Recently, single‐cell RNA sequencing (seq) of ∼46 000 cells from 25 ascites, two primary HGSOC tumors, and three ascites‐derived xenografts reported significant variability in composition and functional programs of HGSOC and suggested that inhibition of the JAK/STAT pathway may have potent antitumor activity.^13^ Another study describing single‐cell RNA profiles of six independent tumors also revealed widespread heterogeneity of HGSOC.^14^ Characterization of the heterogeneous signatures within HGSOC will certainly lead to better understanding of mechanisms for ovarian carcinogenesis, metastasis, and drug resistance.

In this study, we analyzed the transcriptomic profiles of 13 571 cells from four paired primary and metastatic HGSOC samples. Our data unveiled a group of aggressive epithelial cells that are potentially hyperproliferative and resistant to chemotherapies. Also, several new tumor cell markers that are critical for ovarian cancer cell growth and migration were identified and experimentally validated. The trajectory analysis revealed the gene expression signatures during cancer progression, providing the first clue to the role of the translational machinery in ovarian cancer metastasis at the single‐cell layer. Moreover, dissection of the gene regulatory networks suggested the JUN signaling pathway as a potential therapeutic target for treatment of ovarian cancer, which was validated using the JUN/AP‐1 inhibitor T‐5224. Lastly, our study revealed that the epithelial‐fibroblast cell communication is critical for ovarian cancer development.

## 2 RESULTS

### 2.1 Single‐cell expression atlas unveils diverse cell types in HGSOC

**FIGURE 1:**
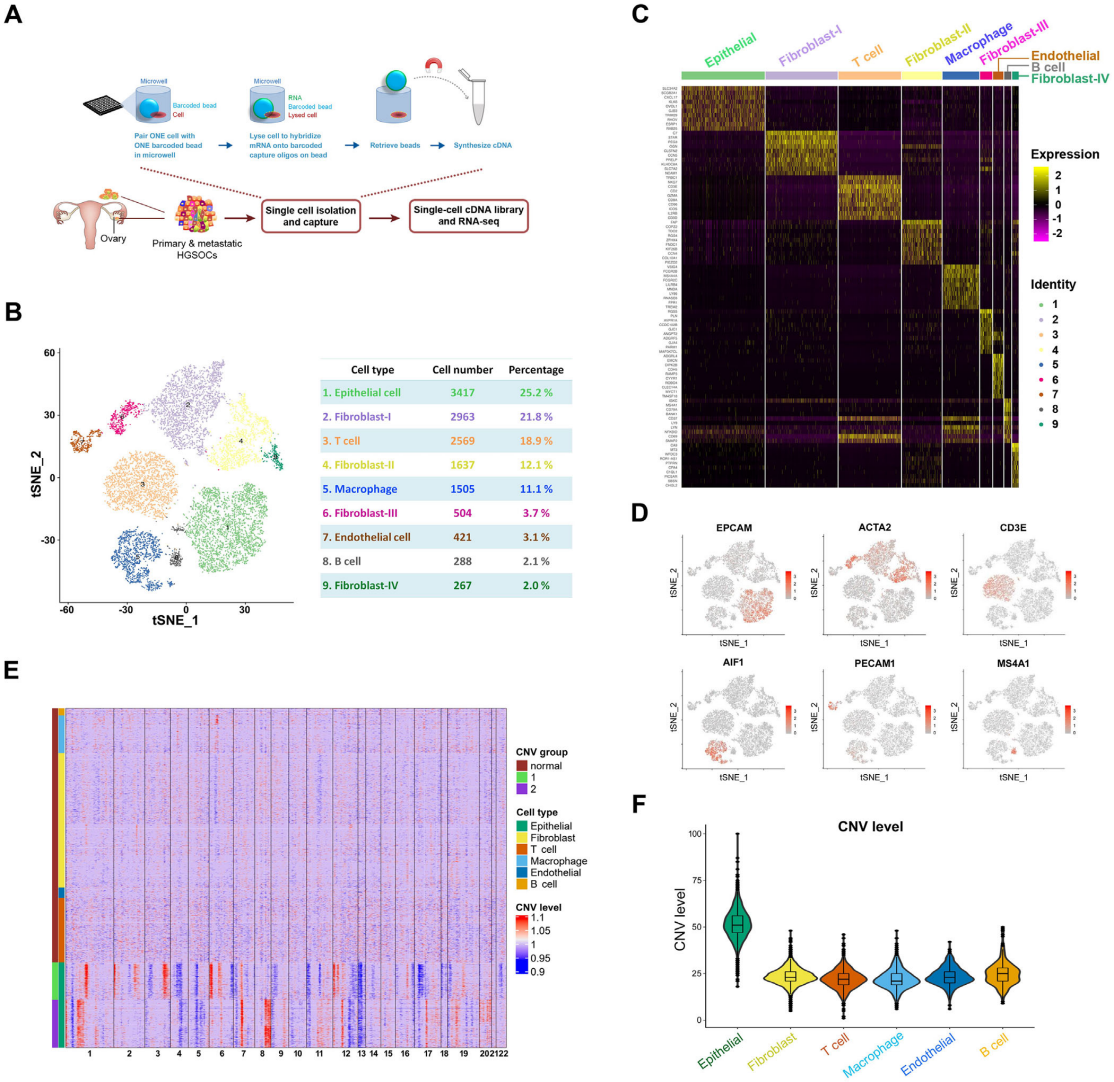
Diverse cell types in HGSOC delineated by single‐cell transcriptomics. (A) Workflow depicting sample collection, single cell preparation, cDNA library construction, and RNA‐sEquation (B) The t‐SNE plot displays main cell types in HGSOC. Cell number and percentage of each cell type are summarized in the right panel. (C) The heatmap displays the top 10 SDE genes in each cell type. (D) Expression levels of specific markers for each cell type are plotted onto the t‐SNE map. Color key from gray to red indicates relative expression levels from low to high. (E) The heatmap displays large‐scale CNVs of epithelial cells. The red color represents high CNV level and blue represents low CNV level. (F) Violin plots show CNV levels among six cell types

To explore intratumoral heterogeneity in HGSOC, we generated single‐cell transcriptome profiles of four matched pair samples of primary and metastatic carcinomas from two patients using nanowells to capture single cells through the BD Rhapsody platform (Figure 1A). After initial quality control, single‐cell transcriptomes in a total of 13 571 cells were acquired for further analysis (Table S1). By analyzing variably expressed genes across all cells, we identified six major cell types in nine clusters of cells, including epithelial cells (cluster 1), fibroblast cells (clusters 2, 4, 6, and 9), T cells (cluster 3), macrophages (cluster 5), endothelial cells (cluster 7), and B cells (cluster 8), by *t*‐distributed stochastic neighbor embedding (t‐SNE) (Figures 1B, S1A‐S1C). We then performed differential gene expression analysis to define the identities of these cell clusters. Each cluster was compared to the other pooled clusters to find unique gene signatures (Table S2) and the top 10 significantly differentially expressed (SDE) genes of each cluster are represented in the heatmap as shown in Figure 1C. The well‐known cell type markers were used to characterize the cell clusters. The epithelial markers EPCAM, KRT5, KRT8, and KRT18 were significantly enriched in the cluster of epithelial cells (Figures 1D and S1D). PAX8 and KRT7, the markers for the fallopian tube epithelium (FTE),^15^ were also selectively expressed in this cluster (Figure S1D), which suggests the FTE origin for HGSOC. The stromal markers, such as DCN, COL6A1, and COL6A2, were highly expressed in the fibroblast cell clusters (Figure S1E). Importantly, this group of fibroblast cells expressed the markers for cancer‐associated fibroblasts (CAFs) including ACTA2 (encoding α‐SMA), PDGFRA, PDGFRB, DDR2, FAP, and CAV1 (Figures 1D and S1E). The immune cells were divided into three distinct clusters, including T lymphocytes, B lymphocytes, and macrophages (Figure 1B). Specific markers were found in each cluster, such as CD3E, CD8A, and CD2 in the T cell cluster (Figures 1D and S1F), MS4A1 (also known as CD20), CD79A and BANK1 in the B cell cluster (Figures 1D and S1I), and AIF1, CD14 and CD163 in the macrophage cluster (Figures 1D and S1G). A small amount of endothelial cells were characterized by their selective expression of markers PECAM1, CDH5, and CD34 (Figures 1D and S1H). Therefore, these results reveal that HGSOC is highly heterogeneous and composed of diverse cell types.

### 2.2 Chromosomal copy number variations identify malignant cells in HGSOC

A plethora of evidence has shown that all tumors consist of transformed malignant cells and nontransformed cells, and the interaction of these two classes of cells creates the tumor microenvironment.^16^ Thus, we sought to define malignant cells among all the six cell types by calculating large‐scale copy number variations (CNVs) inferred from single‐cell gene expression profiles using an approach described previously.^17^ As expected, the epithelial cell cluster showed dramatically higher CNV level compared with other cell types (Figure 1E, F), which is also reflected by the t‐SNE plot (Figure S1J). Interestingly, no significant difference of the CNV levels was observed between the primary and metastatic tumors (Figure S1K).

### 2.3 Distinct molecular features of epithelial cells in HGSOC

**FIGURE 2:**
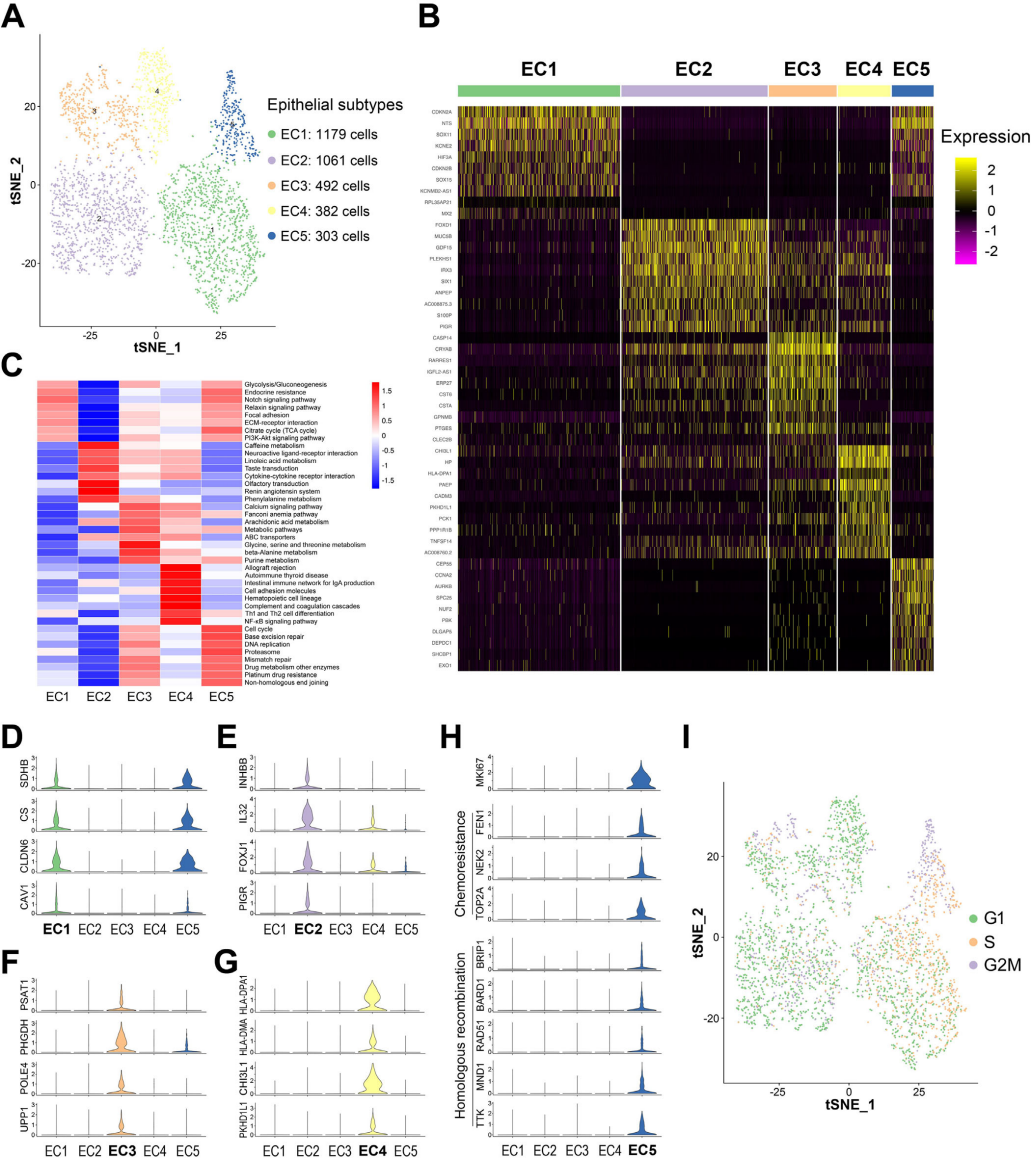
Epithelial cell subtypes reveal distinct molecular features. (A) The t‐SNE plot displays five subclusters of epithelial cells. (B) The heatmap shows the top 10 SDE genes in each subset of epithelial cells. (C) GSVA analysis indicates enriched pathways of each subset of epithelial cells. (D‐H) Violin plots show the expression of selected markers in each subset of epithelial cells. (I) The t‐SNE plot shows epithelial cell subsets in different phases of the cell cycle

To depict their intrinsic portraits, these malignant epithelial cells were further divided into five subclusters (epithelial cluster EC1 to EC5) by the SNN algorithm and t‐SNE analysis (Figures 2A and S2A, S2B). Epithelial cells from the primary and metastatic tumors were separately distributed base on their gene expression signatures (Figure S2C), although they did not show any significant differences in the CNV levels (Figure S2D, S2E). The unique gene signatures and the top 10 SDE genes of each epithelial subgroup were delineated (Table S3 and Figure 2B). Additionally, the Gene Set Variation Analysis (GSVA) was performed to functionally annotate the epithelial subgroups (Figure 2C). EC1 showed gene enrichment for glycolysis/gluconeogenesis, citrate cycle, extracellular matrix (ECM)‐receptor interaction, and focal adhesion, partially sharing a subset of SDE genes with EC5 (Figure 2C, D). The SDE genes of EC2 were involved in the cytokine‐cytokine receptor interaction, and the neuroactive‐related pathways including neuroactive ligand‐receptor interaction, caffeine metabolism, taste transduction, and olfactory transduction (Figures 2C, E and S2F). It has been shown that FTE consists mainly of secretory and ciliated epithelial cell types.^18^ EC2 was found to strongly express the well‐documented ciliated epithelial markers FOXJ1 and PIGR,^19^, ^20^ and several newly identified FTE ciliated markers, such as CAPS and GDF15^15^ (Figures 2E and S2F), suggesting that this group of malignant epithelial cells may originate from ciliated cells, while other subgroups are likely to originate from secretory cells, of FTE. EC3 exhibited higher expression of genes associated with nucleotide and amino acid metabolism. Also, genes involved in the Fanconi anemia pathway and ABC transporters were elevated in EC3, suggesting a potential drug‐resistant feature of this subcluster (Figure 2C, F). EC4 was characterized by the immune response‐related pathways and the complement and coagulation cascades (Figures 2C, G and S2G). The major histocompatibility complex (MHC) class II genes including HLA‐DMA, HLA‐DPA1, HLA‐DPB1, HLA‐DRA and HLA‐DRB1, as well as the MHC II complex‐associated CD74, were also found to be overexpressed in EC4 (Figures 2G and S2G), indicating this group of cells are active in antigen processing and presentation for immune response. Interestingly, EC5, a small proportion (8.9 %) of epithelial cells, displayed significant and remarkable enrichment for pathways associated with cell cycle, DNA replication, DNA repair, and drug metabolism (Figures 2C, H and S2H). The proliferation marker MKI67 and chemoresistance‐associated genes, such as FEN1, NEK2, and TOP2A, were highly overexpressed in this group of cells. In addition, several homologous recombination‐associated genes, such as BRIP1, BARD1, RAD51, MND1, TTK, RAD51AP1, BIRC5, RPA1, and HJURP, were also activated (Figures 2H and S2H), suggesting that these cells may be resistant to PARP inhibitors. Furthermore, we defined the cell cycle stages of epithelial cells according to expression of the S and G2/M phase markers^21^ across all five subclusters, as cell cycle progression is a major indicator of proliferative potential of cancer cells. Consistently, we found that EC5 is mainly composed of S‐phase and G2/M‐phase cancer cells (Figure 2I), again indicating that these cells are hyperproliferative. Altogether, epithelial cells of HGSOC are categorized into five subclusters with distinct functions based on single‐cell gene expression profiles, and EC5 may represent the most aggressive epithelial subtype with enhanced proliferative potential and therapeutic resistance.

We also performed immunohistochemistry (IHC) staining of the EC2 markers MUC5B and FOXD1 and the EC4 markers PAEP and HP in primary and metastatic ovarian tumor samples. Remarkably, we showed that MUC5B‐ or FOXD1‐positive cells are specifically present in primary sites (Figure S2I), while PAEP‐ or HP‐positive cells are exclusively in metastatic sites (Figure S2J). These observations are in line with the former result (Figure S2C), and clearly demonstrate the differences of the cell populations in primary and metastatic ovarian tumors.

**FIGURE 3:**
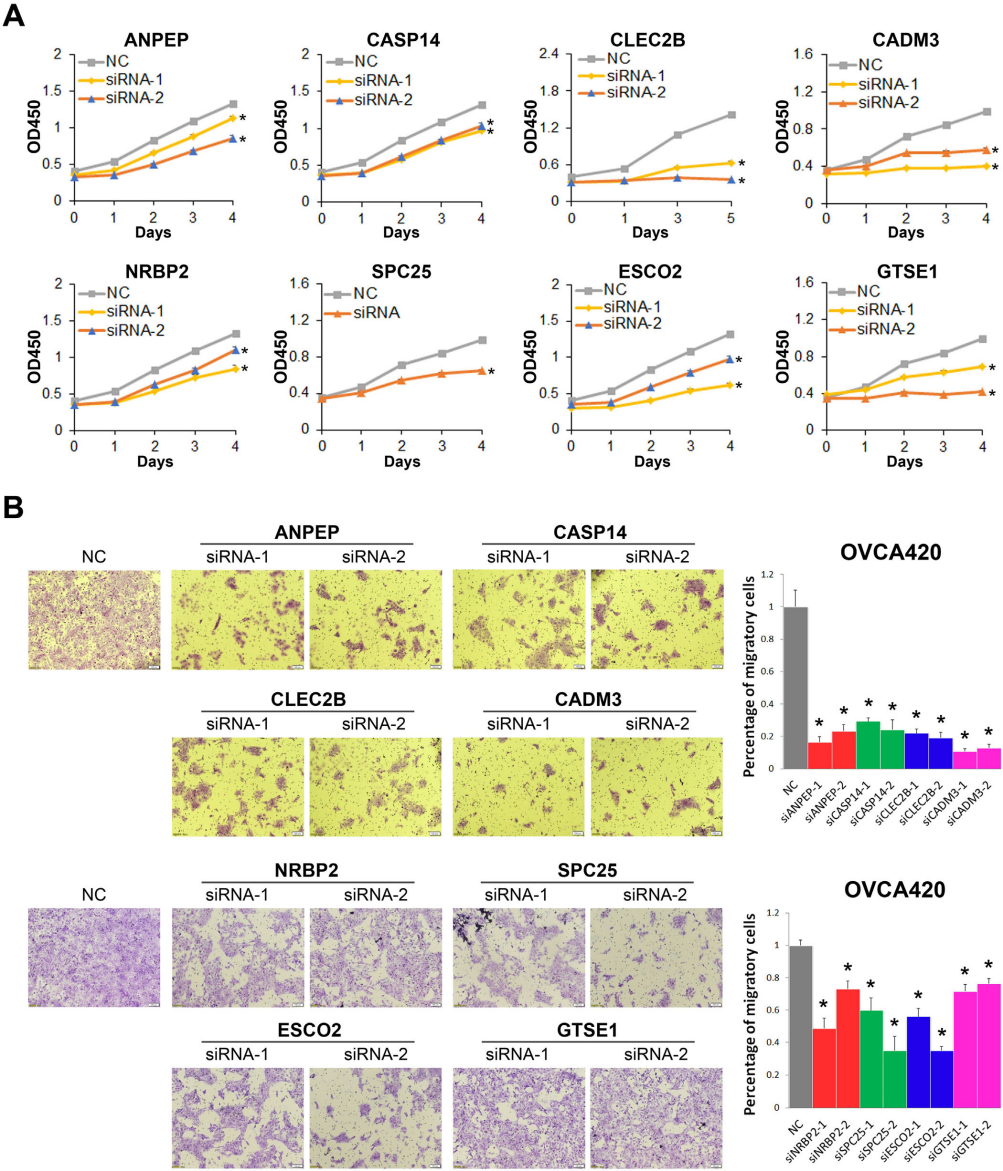
Functional validation of the epithelial markers. (A) Ablation of the newly identified marker genes, including ANPEP, CASP14, CLEC2B, CADM3, NRBP2, SPC25, ESCO2, and GTSE1, inhibits proliferation of ovarian cancer OVCA420 cells. (B) Ablation of any of the newly identified marker genes inhibits migration of ovarian cancer OVCA420 cells. *P < .05 by two‐tailed *t*‐test

Intriguingly, a number of novel markers that may contribute to ovarian cancer development have been identified among the top 25 SDE genes of each subcluster (Figure S2K and Table S3). Particularly, we examined if ANPEP (from EC2), CASP14 and CLEC2B (from EC3), CADM3 and NRBP2 (from EC4), and SPC25, ESCO2, and GTSE1 (from EC5) are responsible for ovarian cancer cell proliferation by the cell viability assay. As shown in Figure 3A, knockdown of each of these genes significantly reduced proliferation of ovarian cancer OVCA420 cells. To elucidate if the eight genes are specific to ovarian cancer, we have performed a set of cell viability assays using the colorectal and breast cancer cell lines, RKO and MCF‐7 (Figure S3A, S3B). Knocking down some of the genes indeed inhibited colorectal or breast cancer cell growth, although the inhibitory effects were mild compared to those in ovarian cancer cells, suggesting that these genes are associated with growth of different cancers, but might be more specific to ovarian cancer. Furthermore, the transwell assay was performed to evaluate if these genes are required for the invasive potential of ovarian cancer cells. Remarkably, depletion of any of the eight genes significantly repressed migration of OVCA420 cells (Figure 3B). Except for ANPEP, also known as CD13, that was shown to be associated with ovarian cancer growth,^22^ the functions of all the other markers during development of ovarian cancer are largely unknown and worthwhile for future investigation.

### 2.4 Dynamic gene expression profiles during progression of HGSOC

**FIGURE 4:**
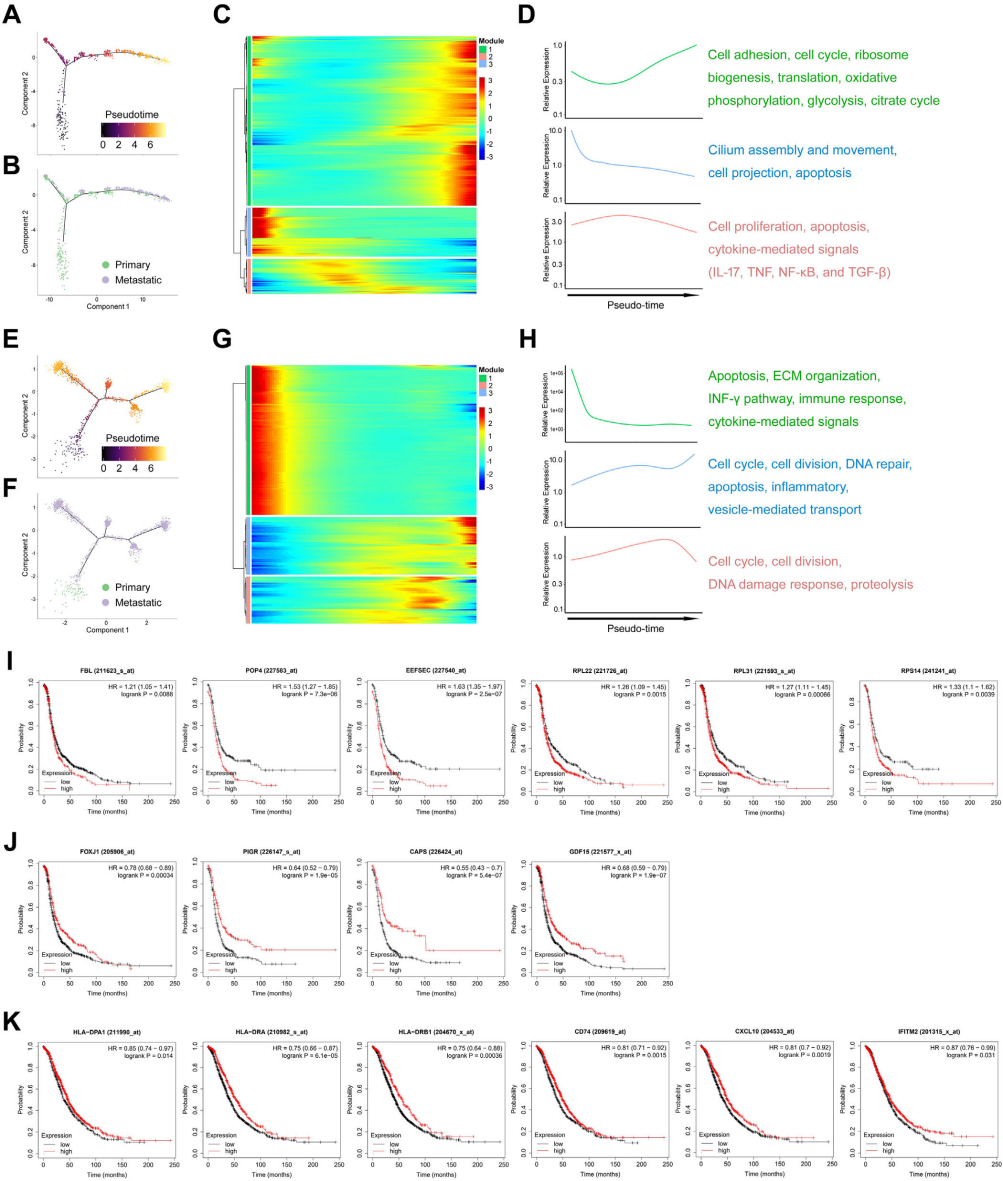
Gene expression profiles during metastasis of HGSOC. (A) The developmental pseudo‐time of epithelial cells (from patient 1) inferred by analysis with Monocle2. Color key from dark to bright indicates cancer progression from the early to the late stage. (B) Primary and metastatic tumor cells are shown in the developmental trajectory. (C) The heatmap displays the SDE genes during progression or metastasis of ovarian cancer. Color key from blue to red indicates relative expression levels from low to high. (D) The GO and KEGG analyses reveals enriched functions and pathways of the SDE genes. (E) The developmental pseudo‐time of epithelial cells (from patient 2) inferred by analysis with Monocle2. Color key from dark to bright indicates cancer progression from the early to the late stage. (F) Primary and metastatic tumor cells are shown in the developmental trajectory. (G) The heatmap displays the SDE genes during progression or metastasis of ovarian cancer. Color key from blue to red indicates relative expression levels from low to high. (H) The GO and KEGG analyses reveals enriched functions and pathways of the SDE genes. (I) Overexpression of ribosome biogenesis‐associated genes predicts poor prognosis in ovarian cancer. (J) Underexpression of ciliated markers predicts poor prognosis in ovarian cancer. (K) Underexpression of immune response‐related genes predicts poor prognosis in ovarian cancer

Pseudo‐time reconstruction was performed to stratify the tumor cells during cancer development. Distribution of epithelial cells from two patients was shown in the trajectory that can predict the de novo tumor progression path (Figure 4A, E). Along the trajectory, the percentage of metastatic epithelial cells increased in the late‐stage cell population (Figure 4B, F), validating the reliability of the trajectory analysis. Our data showed dynamic gene expression profiles during the malignant evolution of tumor cells (Figure 4C, G; Tables S4 and S5). The Gene Ontology (GO) and Kyoto Encyclopedia of Genes and Genomes (KEGG) analyses of these dynamically expressed genes indicated that cell cycle and division, DNA repair, inflammatory, and metabolic pathways, particularly, glycolysis and citrate cycle, are remarkably dysregulated during ovarian cancer progression (Figure 4D, H). Of note, the expression of several positive regulators of cell cycle progression was found to be increased or fluctuated, while the negative regulators were downregulated, suggesting dynamic expression of cell cycle genes is critical for fine‐tuning the cell cycle during ovarian cancer progression (Figure 4D, H). Interestingly, we noticed that a number of ribosome biogenesis and translation‐related genes are upregulated during tumor cell evolution (Figure 4D). Although enlarged nucleoli and increased ribosome biogenesis are features of some cancers, our results at the single‐cell level revealed that enhancement of ribosome biogenesis and protein translation is associated with metastasis of ovarian cancer, which is consistent with previous studies in bulk tumors.^23^, ^24^ Indeed, higher expression of some ribosome biogenesis‐associated genes was significantly correlated with poor prognosis in ovarian cancer (Figure 4I). These results also suggested that the nucleolar stress‐based targeting therapy, such as Actinomycin D and CX‐5461,^25^, ^26^ might be potentially effective in treating metastatic ovarian cancer. On the contrary, multiple signaling pathways were found to be downregulated during ovarian cancer progression. For instance, the expression of the ciliated epithelial markers, including FOXJ1, PIGR, CAPS, and GDF15, declined during the process of metastasis (Figure 4D), although they were overexpressed in EC2 that mainly consisted of primary tumor cells (Figures 2E and S2C, S2F). Consistently, underexpression of these markers predicted worse prognosis in ovarian cancer (Figure 4J). Also, we found that the progression of ovarian cancer is accompanied by reduced expression of the immune response‐related genes (Figure 4H), whose downregulation is significantly associated with unfavorable prognosis (Figure 4K). Moreover, the prognostic value of all the SDE genes was evaluated, and the results revealed that most of these genes are significantly correlated with cancer progression and may serve as prognostic markers (Tables S4 and S5). Together, these results demonstrate dynamic gene expression profiling during ovarian cancer development, and suggest that dysregulation of several critical pathways is associated with prognosis of ovarian cancer.

### 2.5 Characterization of fibroblasts in HGSOC

**FIGURE 5:**
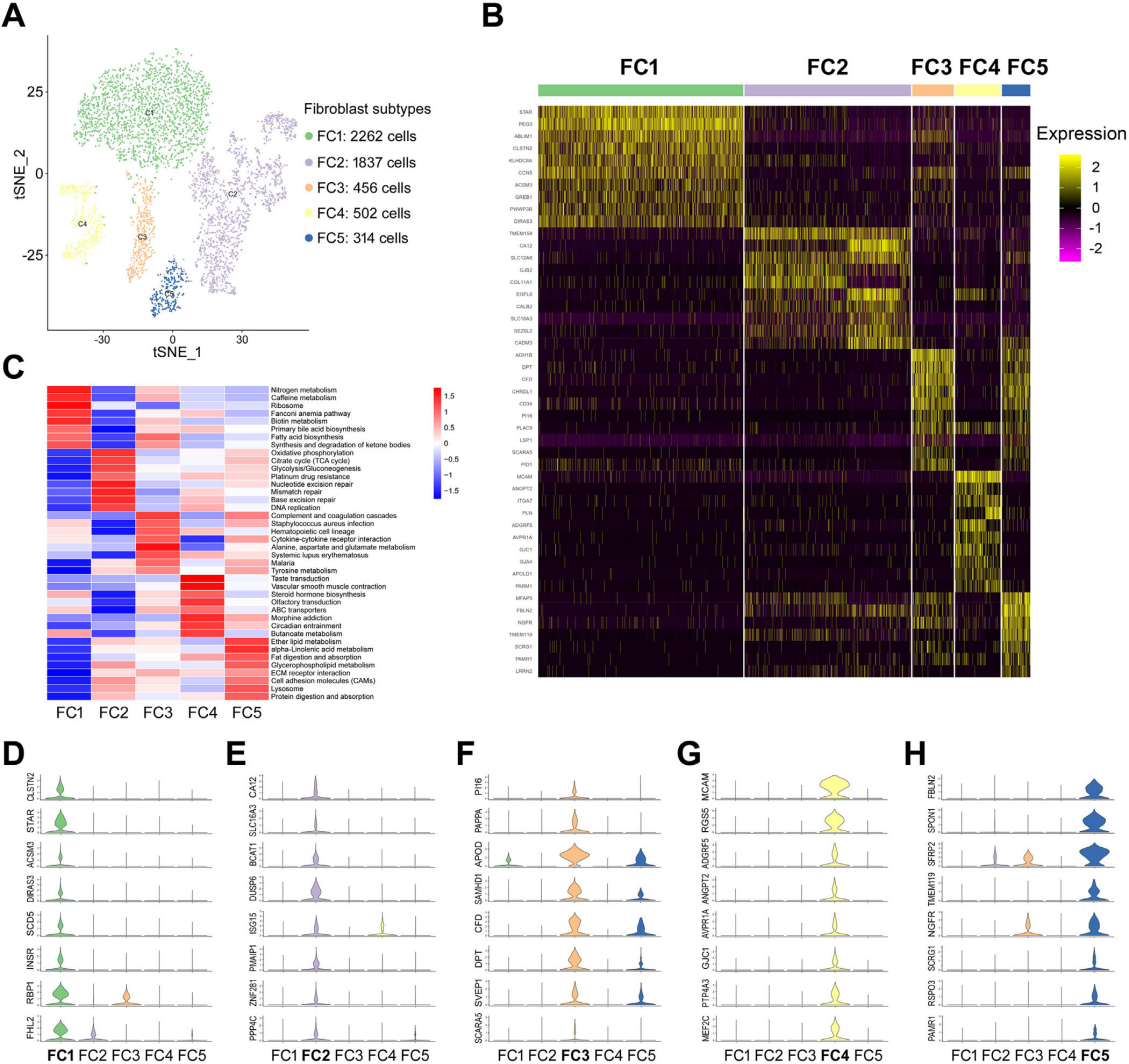
Distinct functions of fibroblast cell subtypes. (A) The t‐SNE plot displays five subclusters of fibroblast cells. (B) The heatmap shows the top 10 SDE genes in each subset of fibroblast cells. (C) GSVA analysis indicates enriched pathways of each subset of fibroblast cells. (D‐H) Violin plots show the expression of selected markers in each subset of fibroblast cells

Fibroblasts have been considered to be a highly abundant and heterogeneous population of cells in tumors. Fibroblast cells from HGSOC were reanalyzed and categorized into five distinct subclusters (fibroblast cluster FC1 to FC5) by t‐SNE analysis (Figures 5A and S4A‐S4C). The unique gene signatures and the top 10 SDE genes associated with each fibroblast subcluster were delineated (Table S6 and Figure 5B). The GSVA analysis was performed to functionally annotate the fibroblast subsets (Figure 5C). FC1 showed gene enrichment primarily for lipid and steroid metabolism (Figure 5C), as evidenced by several marker genes, such as ACSM3, STAR, SCD5, and others (Figure 5D). FC2 exhibited preference for genes involved in glucose metabolism, including glycolysis/gluconeogenesis, citrate cycle, and oxidative phosphorylation, and multiple DNA repair pathways (Figure 5C), which was validated by several representative markers as shown in Figure 5E. Interestingly, FC2 displayed the highest correlation with epithelial cells (Figure S4D) and showed specific expression of tumor epithelial markers KRT8, KRT10, and KRT18 (Figure S4E), as well as the EMT markers SNAI1 and SNAI2 (Figure S4E), suggesting that FC2 may have the tumor‐like aggressive potential. The observations indicate that these cells may represent the aggressive population of fibroblasts and may confer tumor resistance to anticancer chemotherapies. Highly expressed genes in FC3 were enriched in the immune response‐related pathways, as they are mainly associated with immune or infectious diseases (Figure 5C, F). Although FC4 showed higher level of genes involved in diverse pathways, such as taste and olfactory transduction, steroid hormone biosynthesis, ABC transporters, etc. (Figure 5C), we particularly observed dramatic upregulation of substantial vascular genes, such as MCAM, RGS5, ADGRF5, and ANGPT2, in this subcluster (Figure 5G), suggesting an essential role of this group of fibroblast cells in vascular development and angiogenesis. FC5 was found to highly express genes enriched in different lipid metabolic pathways and the extracellular matrix signaling (Figures 5C). Additionally, a subset of genes associated with development, tissue regeneration, and stem‐like features were also elevated in the subcluster (Figure 5H). Finally, we performed IHC staining of the FC1 marker STAR and the FC5 marker MFAP5 in primary and metastatic ovarian tumor samples. As expected, STAR expressed at a higher level in primary fibroblasts compared to metastatic fibroblasts (Figure S4F), while MFAP5 exhibited a higher expression level in metastatic fibroblasts than primary fibroblasts (Figure S4G), which was consistent with the result illustrated in Figure S4C. These results unveil the heterogeneity of HGSOC fibroblasts that can support ovarian cancer development by promoting chemoresistance, immune‐suppression, angiogenesis, and tumor cell migration.

### 2.6 Gene regulatory networks identify the JUN pathway as a driver for HGSOC

**FIGURE 6:**
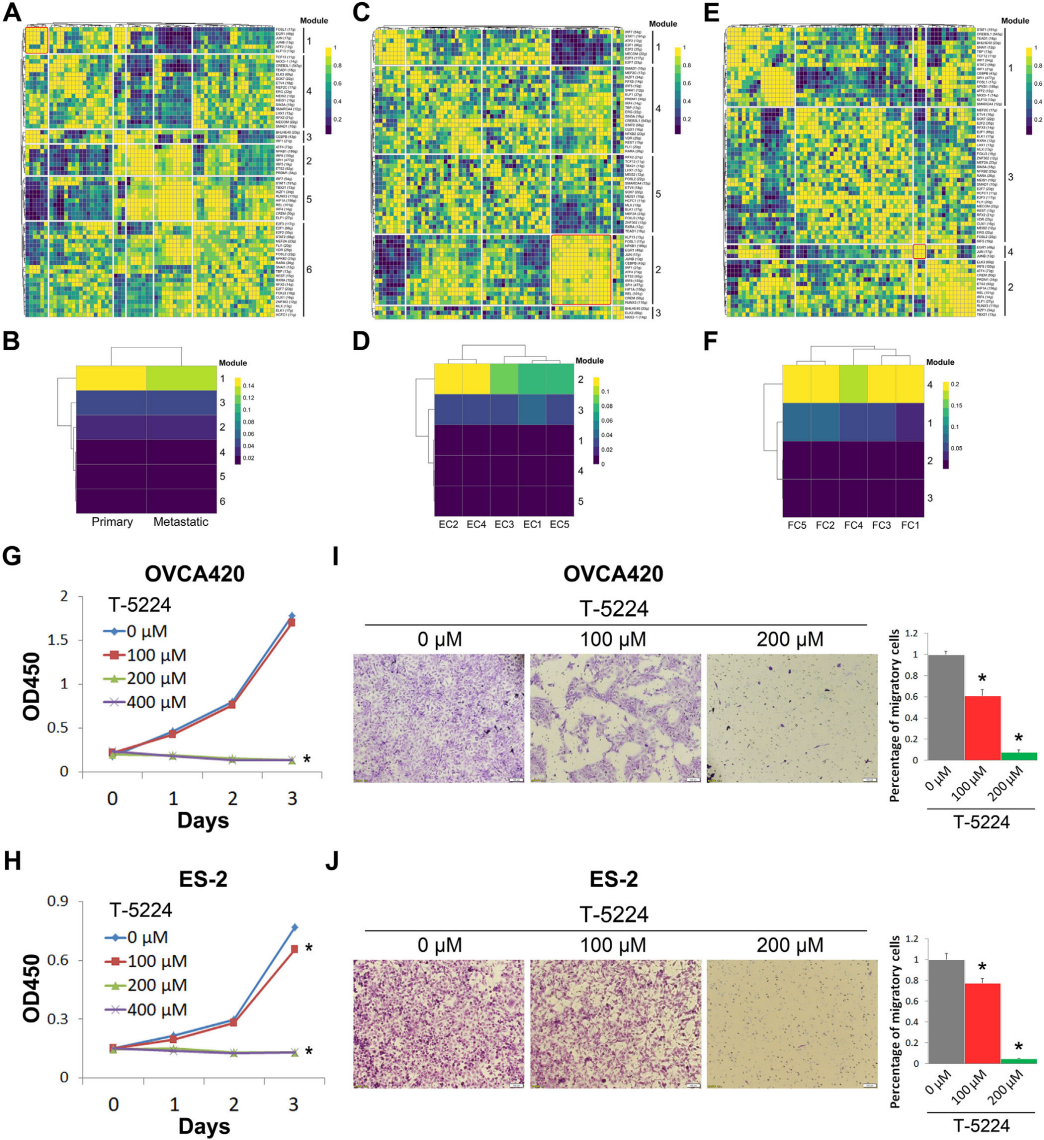
Gene regulatory networks in HGSOC. (A, B) The CSI matrix highlights regulon‐to‐regulon correlation across all cell types from primary and metastatic tumors. Hierarchical clustering of regulons identifies six distinct regulon modules (A). The heatmap shows the regulation activity of each module (B). Color key from blue to yellow indicates the levels of activity from low to high. (C, D) The CSI matrix highlights regulon‐to‐regulon correlation across five epithelial cell subtypes. Hierarchical clustering of regulons identifies five distinct regulon modules (C). The heatmap shows the regulation activity of each module (D). Color key from blue to yellow indicates the levels of activity from low to high. (E, F) The CSI matrix highlights regulon‐to‐regulon correlation across five fibroblast cell subtypes. Hierarchical clustering of regulons identifies four distinct regulon modules (E). The heatmap shows the regulation activity of each module (F). Color key from blue to yellow indicates the levels of activity from low to high. (G, H) The JUN/AP‐1 inhibitor T‐5224 suppresses proliferation of ovarian cancer cell lines OVCA420 (G) and ES‐2 (H). (I, J) The JUN/AP‐1 inhibitor T‐5224 suppresses migration of ovarian cancer cell lines OVCA420 (I) and ES‐2 (J). *P < .05 by two‐tailed *t*‐test

Transcription factors (TFs) and their downstream‐regulated genes constitute a complex and intermingled network of gene regulation, which determines and maintains cell identity. Single‐cell regulatory network inference and clustering (SCENIC) analysis was performed to infer the activity of regulons (a TF together with its target genes comprise a regulon)^27^ for the primary and metastatic cell types (Figure 6A, B), epithelial cell clusters (Figure 6C, D), and fibroblast cell clusters (Figure 6E, F), respectively. The regulon modules based on the regulon crosstalk (regulon‐to‐regulon correlation) were determined by the Connection Specificity Index (CSI) that ranks the regulon significance and mitigates the effects of nonspecific interactions. The analysis of the primary and metastatic groups led to 65 regulons across six regulon modules (Figure 6A). Comparison of the activity of the six modules revealed that the module 1, including the TFs FOSL1, EGR1, JUN, JUNB, ATF2, and KLF13, displays the highest regulation activity in both primary and metastatic cell types (Figure 6B). The analysis of the epithelial clusters revealed that the module 2 has the highest regulation activity among the five regulon modules (Figure 6C, D). Along with the TFs NF‐κB, C/EBPβ, ETS2, and HIF1A that have been well‐documented as tumor promoters, JUN and its 17 downstream target genes were also categorized in this highly active module (Figure 6C). Consistently, the JUN signaling pathway was also identified as one of the most active regulons across the fibroblast clusters (Figure 6E, F). These results suggested that JUN may act as an essential driver for ovarian carcinogenesis. JUN is a component of the activator protein‐1 (AP‐1) complex by forming homodimers or heterodimers with members of JUN, FOS, or ATF family.^28^ Interestingly, JUNB, FOSL1, ATF2, and ATF4, which can form heterodimers with JUN, were also identified in these active modules (Figure 6A, C, E). These findings prompted us to test if inhibition of the AP‐1/JUN signaling pathway suppresses ovarian cancer cell growth. We then tested the idea by using the JUN/AP‐1 inhibitor T‐5224 that was originally developed for arthritis treatment by selectively impeding transcriptional activity of c‐Jun and c‐Fos.^29^ The results indicated that treatment of OVCA420 and ES‐2 cells with T‐5224 significantly inhibits their survival and proliferation in a dose‐dependent fashion (Figure 6G, H). Interestingly, T‐5224 also has a growth‐inhibitory effect on normal ovarian epithelial IOSE cells, though to a less extent compared to ovarian cancer cells (Figure S5). This is probably because c‐Jun is also moderately expressed in ovary,^30^, ^31^, ^32^ and associated with differentiation of the normal ovarian surface epithelium.^33^ Furthermore, we found that T‐5224 can effectively and dose‐dependently suppress ovarian cancer cell migration by the transwell assay (Figure 6I, J). It should be noted that the inhibition of cell migration by 200 μM T‐5224 might be partially because of reduced cell survival. Together, our results suggest the JUN signaling pathway as a potential therapeutic target for ovarian cancer.

### 2.7 Intercellular epithelial‐fibroblast cell communication in HGSOC

**FIGURE 7:**
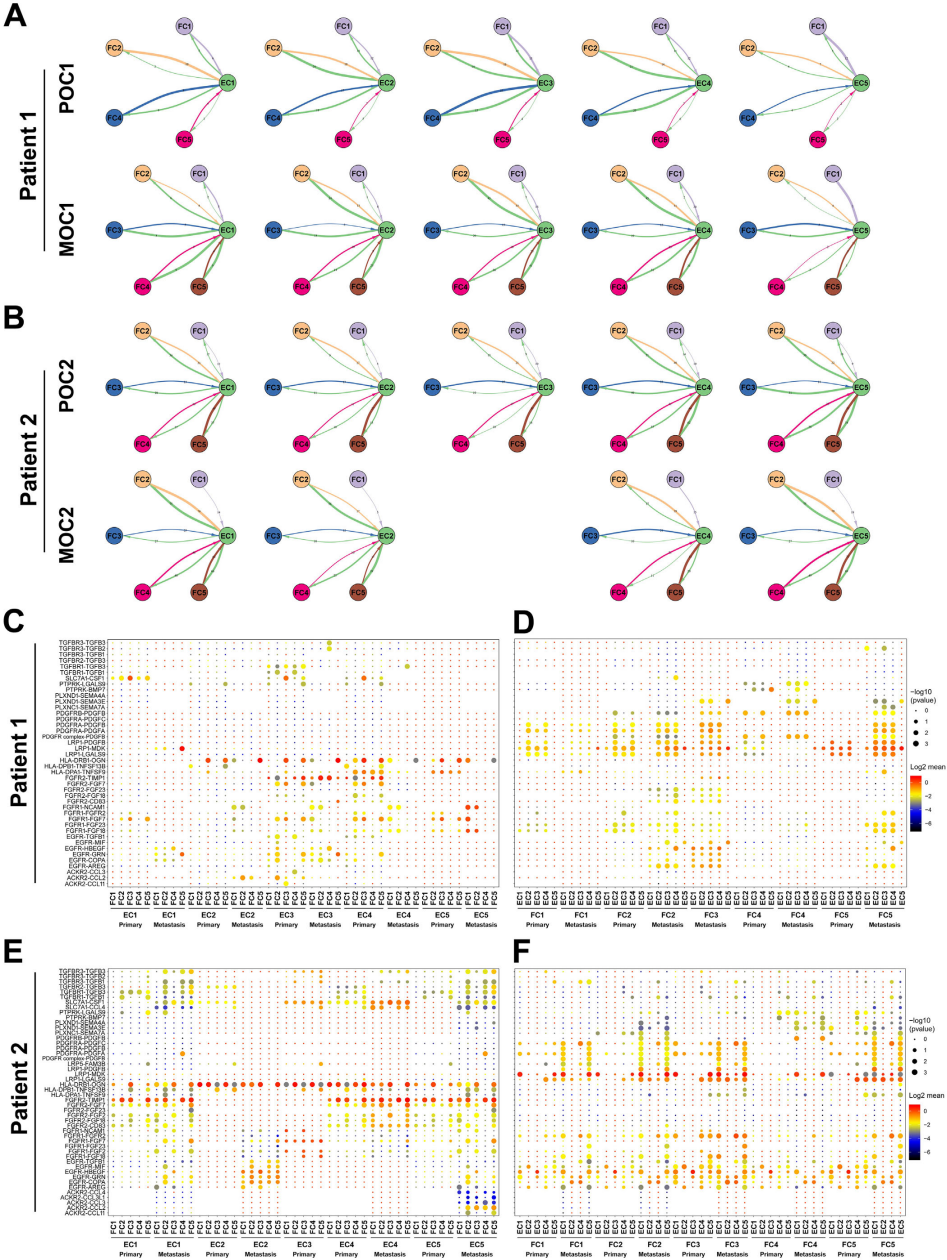
Intercellular epithelial‐fibroblast cell communication in HGSOC. (A, B) Detailed view of ligand‐receptor connections between each epithelial cell subcluster and five fibroblast cell subclusters. Each color arrowline indicates the ligands to receptors from one cell population to another. The arrowline thickness is proportional to the number of ligand‐receptor pairs. (C, E) Overview of selected interactions of receptors expressed by epithelial cells and ligands expressed by fibroblast cells. (D, F) Overview of selected interactions of receptors expressed by fibroblast cells and ligands expressed by epithelial cells

Since the crosstalk between tumor and fibroblast cells in the tumor microenvironment is critically involved in cancer progression,^34^ we characterized the intercellular receptor‐ligand pairs and molecular interactions of the two cell types by the CellPhoneDB algorithm.^35^ Interestingly, we found that the frequency of receptor‐ligand interactions between epithelial and fibroblast cells varies markedly when comparing the primary with the metastatic tumors (Figure 7A, B). Specifically, our analyses further identified diverse receptor‐ligand interplays in both patients. Tumor cells expressed relatively high levels of EGFR, FGFR1, and FGFR2, while their corresponding ligands, such as COPA, GRN, HBEGF, FGF2, FGF7, and FGF18, were widely expressed in fibroblast cells (Figure 7C, E). Notably, the FGFR2‐TIMP1 and HLA‐DRB1‐OGN complexes were likely the most active receptor‐ligand interactions between epithelial and fibroblast cells in both patients. The EGFR‐COPA/GRN/HBEGF pairs were expressed at higher levels in the metastatic tumors than those in the primary tumors, suggesting that these cell‐to‐cell connections might be important for ovarian cancer metastasis. As expected, although many receptor‐ligand pairs were identical in both patients, some of them did show different patterns of interaction between distinct cell subclusters (Figure 7C, E), revealing intertumoral heterogeneity between the two patients. While fibroblast cells, like tumor cells, expressed EGFR and FGFR1, they also exclusively expressed higher levels of the receptors LRP1, PDGFRs, and PLXND1 in both patients (Figure 7D, F). Correspondingly, epithelial cells expressed their ligands, LGALS9/MDK, PDGFs, and SEMAs (semaphorins) (Figure 7D, F). These results indicate that the crosstalk between tumor cells and fibroblast cells via diverse receptor‐ligand signals may exert a profound effect on ovarian cancer development and metastasis.

## 3 DISCUSSION

HGSOC is characterized by abdominal metastasis, high risk of relapse, and chemoresistance. Single‐cell RNA‐seq opens an avenue to better understand the fundamental mechanisms of this disease. In line with previous studies,^13^, ^14^ our results revealed vast intratumoral heterogeneity of HGSOC by identifying diverse cell types, including epithelial cells, fibroblast cells, T cells, B cells, macrophage, and endothelial cells. Through further clustering of the epithelial cells, we particularly identified a subset of epithelial cells (EC5) highly expressing the proliferation marker Ki‐67, and substantial genes associated with DNA repair pathways and chemoresistance (Figure 2C, H and S2H). PARP inhibition is a targeted strategy to induce synthetic lethality in tumors, such as HGSOC, with BRCA1/2 mutations or homologous recombination deficiency (HRD).^36^, ^37^ Intriguingly, these cells also overexpressed a myriad of homologous recombination‐associated genes, strongly suggesting that they are active in the homologous recombination pathway leading to resistance to PARP inhibition. Previous studies indicate that epithelial ovarian cancer can originate from the FTE that is mainly composed of secretory and ciliated epithelial cell types.^18^ We found that the EC2 cluster of epithelial cells is enriched for ciliated epithelial markers, including FOXJ1, PIGR, CAPS, and GDF15 (Figures 2E and S2F). This observation verifies the FTE as a common origin of ovarian cancer and, as a complement to the previous knowledge,^15^ indicates that HGSOC tumor cells also preserve some ciliated cell characteristics of its progenitor lineage. Additionally, our study identified a number of novel markers, such as CASP14, CLEC2B, CADM3, NRBP2, SPC25, ESCO2, and GTSE1, which are upregulated in a cluster‐specific manner (Figure S2K) and critical for ovarian cancer cell proliferation and migration (Figure 3A, B). In line with previous studies,^38^ we also showed that malignant epithelial cells are clustered largely according to their tumor of origin because of the profound intertumoral heterogeneity. Hence, analysis of more tumor samples would be helpful to unveil the vast diversity of mechanisms and, as thus, to individualized treatment of this cancer. Fibroblasts are the most abundant population in the tumor microenvironment critical for tumor angiogenesis, metastasis, and drug resistance.^34^ Indeed, our data unveiled multiple sub‐populations of fibroblasts that are involved in the above processes. Importantly, a subset of possible malignant fibroblast cells (FC2) was identified, as they highly expressed genes enriched in the metabolic pathways, DNA replication and repair, and the platinum drug resistance pathways (Figure 5C, E). Consistently, we found that these fibroblast cells are closely correlated with epithelial cells (Figure S4D), as they highly expressed the epithelial markers, such as KRT8, KRT10, and KRT18 (Figure S4E). Altogether, our results provide new evidence by single‐cell transcriptomics for the phenomenon that a small portion of epithelial and fibroblast cells are critical for ovarian cancer development and drug resistance.

To dissect the molecular features during progression of ovarian cancer, we conducted the trajectory analysis of the single‐cell RNA‐seq data, which allows us to identify the gene expression profiles along the developmental path of cancer. The pseudo‐time reconstruction model was proved to be robust and reliable, as the tumor cells distributed along the progression trajectory in the primary‐to‐metastasis pattern (Figure 4A, B, E, F). We found that the signaling pathways including cell cycle and division, DNA repair, inflammation, and energy metabolism that are highly associated with tumor growth, metastasis, and drug resistance are markedly dysregulated during ovarian cancer progression (Figure 4D, H). Importantly, global upregulation of the ribosome biogenesis and translation‐related genes were found to be accompanied by cancer metastasis (Figure 4D; Tables S4 and S5), which is in line with a recent work that showed the first link of ribosomal proteins to cancer metastasis in a metastatic mouse model of breast cancer.^39^ Ribosome biogenesis is a tightly organized multistep process, including synthesis and processing of ribosomal RNAs (rRNAs), production of ribosomal proteins (RPs), and consequent assembly and maturation of the ribosome.^40^ Given that the tumor cells require highly motivated ribosome biogenesis and protein synthesis to boost their own growth and proliferation, targeting the translational machinery has emerged as a potent therapeutic approach for cancer treatment.^25^ It has been well‐established that perturbation of any single step of ribosome biogenesis by, for example, Actinomycin D^41^, ^42^ or CX‐5461,^26^, ^43^ triggers a drastic cytotoxic effect, namely ribosomal stress or nucleolar stress, unleashing tumor suppressive signals involving activation of the tumor suppressors p53 and TAp73,^44^, ^45^, ^46^ and inhibition of the c‐Myc pathway.^25^, ^47^, ^48^ Together with these findings, our results strongly suggest that inhibition of ribosome biogenesis could be a promising strategy for treatment of metastatic HGSOC.

Gene regulatory networks can be reflected by the regulation of a number of TFs and their downstream target genes, which are the molecular basis underlying cell states. To determine the robust gene regulatory networks and the potential critical TFs that drive HGSOC, we conducted SCENIC analysis of the single‐cell RNA‐seq data. Three most active regulon modules that may be important for cell state maintenance and cancer development were identified by comparing the primary and metastatic cell types (Figure 6A, B), five epithelial cell clusters (Figure 6C, D), and five fibroblast cell clusters (Figure 6E, F), respectively. Intriguingly, only the JUN signaling pathway was consistently found in all these three active modules, suggesting this TF as a potential driver for ovarian cancer. JUN is a multifaceted TF as a component of the transcription factor AP‐1 complex whose function is associated with embryonic development, tissue‐specific development, such as neuron, blood and bone, T cell differentiation and activation, and cancer cell survival and proliferation.^49^ Of clinical importance, we validated that inhibition of JUN drastically suppresses ovarian cancer cell growth and migration by employing the JUN/AP‐1 inhibitor T‐5224 that was used for treatment of inflammatory disorders (Figure 6G‐J),^29^ providing the first line of evidence of JUN as a potential druggable target in the treatment of ovarian cancer. Nevertheless, tumor xenograft models fostering a heterogeneous tumor microenvironment may be required for testing the antitumor effect of JUN inhibition, as the JUN activity is also elevated in tumor fibroblasts (Figure 6E, F).

By dissecting the signaling network of epithelial‐fibroblast cell communication, we identified several receptor‐ligand complexes that should be critically important for ovarian cancer development (Figure 7C‐F). For instance, the FGFR‐FGF signals were shown to promote cancer cell growth, differentiation, and motility,^50^ while the PDGFR‐PDGF signals are particularly essential for mesenchymal cell proliferation, survival, and migration.^51^ The ACKR2‐CCL2 interaction was observed in our study, which is in line with previous work showing that ACKR2‐CCL2 is associated with lymphatic vascular development^52^ and melanoma and breast cancer metastasis.^53^ Interestingly, a number of underappreciated receptor‐ligand pairs were also uncovered, such as HLA‐DRB1‐OGN, SLC7A1‐CSF1, LRP1‐LGALS9, and LRP1‐MDK.

In conclusion, this study demonstrates the intratumoral heterogeneity of HGSOC by single‐cell RNA transcriptomics analysis. Several novel markers, prognostic factors, and the potential therapeutic target have been identified and validated. Additionally, our data have depicted the gene regulatory signaling networks and the epithelial‐fibroblast cell communication in HGSOC. Thus, our study provides a new angle on understanding HGSOC and the JUN pathway as a potential new target for future development of anti‐HGSOC therapy.

## 4 MATERIALS AND METHODS

### 4.1 Preparation of single‐cell suspensions from HGSOC samples

Two HGSOC patients aged 53 and 57, who were treatment‐naïve before surgery, donated two pairs of matched primary and metastatic tumor samples. Specifically, metastases were collected from omentum. This study was approved by the Ethics Committee of Fudan University Shanghai Cancer Center. Informed consent was obtained from the patients. More than ten thousand cells were captured through a limited dilution approach. The surface of supersaturated beads was filled with oligonucleotide barcodes, thus a bead could be paired with a cell in a microwell. Once cells lysed in the cell lysis buffer, the beads were hybridized by polyadenylated RNA molecules, which was then used for reverse transcription. Each cDNA was tagged with special cell label fragment during cDNA synthesis to indicate the cell of origin. Library for scRNA‐seq were generated using BD Rhapsody platform and sequenced on the Illumina Novaseq 6000.

### 4.2 Single‐cell RNA seq data preprocessing

After sequencing, the analysis pipeline takes the FASTQ files, a reference genome file and a transcriptome annotation file for sequence alignment. The pipeline generates a UMI count matrix which will be processed using Seurat^54^ software (version 3.1.1) for further analysis. The low‐quality cells and likely multiple microdroplets were removed. To do so, we filtered out cells with UMI/gene numbers outside of the mean value ±2 fold of SD. The low‐quality cells containing >50% of the counts from mitochondrial genes were discarded. After the quality control, 13 571 single cells in total remained and were subjected to the following analyses. The normalization of the library size was carried out using the NormalizeData function^54^ to obtain the normalized counts. Specifically, gene expression measurements for every single cell were LogNormalize normalized, multiplied by a scaling factor (10 000 by default), and the results were logtransformed.

Highly expressed genes across single cells were identified by the previously described method.^55^ The most variable genes were selected employing the FindVariableGenes function in Seurat.^54^ To remove the batch effects in single‐cell RNA seq data, the mutual nearest neighbor (MNN) was performed as described.^56^ The shared nearest neighbor (SNN) algorithm was used for clustering, which is the default algorithm for clustering in the pipeline of Seurat. It includes two steps corresponding to the two functions. First, Findneighbors was used to calculate the K‐nearest neighbors (KNN) of each cell and construct the SNN graph image. Second, Findclusters was used to find cell clusters according to the SNN graph results (so it is called graph‐based clustering). All parameters used were default parameters. Cells were reclustered separately without engaging all the other cell types. After clustered based on their gene expression patterns employing the FindClusters function, cells were then visualized with the RunTSNE function in Seurat.^54^ We used the FindAllMarkers function (test.use = bimod) in Seurat to identify marker genes of each cluster.^54^ For a given cluster, positive markers were identified by the FindAllMarkers function compared with all other cells.

The FindMarkers function in Seurat was employed to identify the differentially expressed genes (DEGs).^54^ The signification threshold was set as *P* < .05 and |log2foldchange| > 0.58. DEG's GO and KEGG analyses were conducted respectively using R based on the hypergeometric distribution. GSVA converted the expression matrix into a path enrichment score matrix, and the different pathways were then obtained through the lmFit analysis of the limma package.

### 4.3 CNV analysis

We used the inferCNV software^38^ to estimate initial CNVs for each region. CNV was calculated based on the expression level for each cell with a cutoff of 0.1. Genes were sorted according to their genomic location, and a moving average of gene expression was determined by the range of 101 genes. By subtracting the mean, the expression was centered to zero. Epithelial cells were considered as malignant cells, while all the other cells were regarded as normal cells. Finally, the de‐noising was conducted to produce the CNV profiles.

### 4.4 Pseudo‐time analysis

The developmental pseudo‐time was determined with the Monocle2 package.^57^ The importCDS function was employed to convert the raw counts from Seurat object into CellDataSet object. The differentialGeneTest function was employed to select ordering genes (qval < 0.01). The dimensional reduction clustering analysis was carried out by using the reduceDimension function, and the orderCells function was then used for trajectory inference. Changes of gene expression were tracked over pseudo‐time by employing the plot_genes_in_pseudotime function.

### 4.5 SCENIC analysis

The SCENIC analysis was conducted utilizing the motifs database for RcisTarget and GRNboost. The softwares of SCENIC^27^ version 1.1.2.2, AUCell 1.4.1, and RcisTarget 1.2.1 were used. In detail, overrepresented transcription factor (TF) binding motifs were identified through the gene list in RcisTarget package. The AUCell package was used to score the activity of each group of regulons in every single cell. The scFunctions (https://github.com/FloWuenne/scFunctions/) package was used to calculate the connection specificity index (CSI) for all the regulons.

### 4.6 Cell‐cell communication analysis

The CellPhoneDB (v2.0)^35^ was employed to identify correlated ligand‐receptor pairs from single‐cell RNA‐seq data as described previously.^58^ If 10% of the cells within a specific cell cluster had nonzero read counts for the gene encoding a ligand or a receptor, the ligand or the receptor was considered as “expressed.” To constitute the networks of cell‐to‐cell communication, we linked any two cell types where the ligand was expressed in the former cell type and the receptor in the latter. Igraph and Circlize softwares were employed to show the networks of cell‐to‐cell communication.

### 4.7 Cell culture, transient transfection, and reagents

Human ovarian cancer cell lines, OVCA420 and ES‐2, used in the study were commercially purchased from American Type Culture Collection. The protocol and condition of cell culture were described previously.^59^ siRNA transfection was conducted using Hieff TransTM Liposomal transfection reagent (Yeasen, Shanghai, China) as described.^59^ The JUN/AP‐1 inhibitor T‐5224 was purchased from MedChemExpress (Shanghai, China). The siRNA sequences used were as follows, siANPEP‐1: GTAAAGCGTGGAATCGTTATT, siANPEP‐2: GGAACCTGGTGACCATAGATT, siCASP14‐1: CGGCAGCTGAGATTCGAAATT, siCASP14‐2: GACATATCTTGGAACTTCTTT, siCLEC2B‐1: GAATTTTCTTAGGCGGTATTT, siCLEC2B‐2: ATACAACTGTTCCACTCAATT, siCADM3‐1: AGATCCACCTCTCAACGCATT, siCADM3‐2: CACTGGTTATAAATCTTCATT, siNRBP2‐1: AGAGATTTCTATGCCCTCATT, siNRBP2‐2: CAGTGCAACCTGGAGAGAATT, siSPC25: GATCGACTTGGACTAGAAATT, siESCO2‐1: CAAAATCGAGTGATCTATATT, siESCO2‐2: GTATCAACCAAAGTATAGATT, siGTSE1‐1: GGAATTAAATAATCCGGTTTT, and siGTSE1‐2: CGGCCTCTGTCAAACATCATT.

### 4.8 Cell viability assay

Cell viability was determined by the Cell Counting Kit‐8 (Dojindo Molecular Technologies, Japan) as described previously.^59^ Cells transfected with siRNAs or treated with T‐5224 were seeded in 96‐well plates. WST‐8 at a final concentration of ten percent was added to cell cultures, and cell viability was assessed by measuring the absorbance at 450 nm every 24 hours for 4‐5 days.

### 4.9 Transwell invasion assay

The cell invasive potential was evaluated using transwell chambers as described previously.^60^ The upper chamber contained cell cultures in serum‐free medium, while the lower chambers were filled with the complete medium. Cells were treated with T‐5224 or DMSO for 48 hours at 37 ℃. The migratory cells reaching to the lower surface of the chamber were fixed and stained in 0.1% crystal violet. The number of invaded cells was quantified by the image J software.

### 4.10 Immunohistochemistry (IHC) staining

The tumor tissues were collected from Fudan University Shanghai Cancer Center. The IHC staining was carried out with the following antibodies, anti‐PAEP antibody (18187‐1‐AP, 1:200 dilution; Proteintech, Rosemont, USA), anti‐MUC5B antibody (28118‐1‐AP, 1:400 dilution; Proteintech, Rosemont, USA), anti‐Haptoglobin antibody (16665‐AP, 1:200 dilution; Proteintech, Rosemont, USA), anti‐STAR antibody (12225‐1‐AP, 1:200 dilution; Proteintech, Rosemont, USA), anti‐MFAP5 antibody (15727‐1‐AP, 1:800 dilution; Proteintech, Rosemont, USA), and anti‐FOXD1 antibody (A20240, 1:200 dilution; ABclonal, Wuhan,China). The immunostaining results were examined by two researchers independently.

### 4.11 TCGA database

Survival of ovarian cancer patients was evaluated by the Kaplan‐Meier method utilizing the KM plotter database (kmplot.com/analysis/).^61^

### 4.12 Statistics

The in vitro experiments were carried out in biological triplicate. The Student's *t*‐test was performed to determine the differences between two groups or more than two groups. * indicates *P* < .05, which is considered statistically significant. Quantitative data are presented as mean ± SD.

## CONFLICT OF INTEREST

The authors declare that they have no conflict of interest.

## AUTHOR CONTRIBUTIONS

Q.H., J.L., Q.Z., and F.X. conducted most of the study and analyzed the data; Q.H. designed and supervised part of the study; J.L. collected the tumor samples; Q.Z. performed the cell viability assay; F.X. conducted immunohistochemistry staining; B.X. analyzed the data in part; H.L. provided instructions and edited the manuscript; X.W. and X.Z. conceived, designed, and supervised the study. X.Z. analyzed the data and wrote the manuscript.

## Supporting information

Supporting Information

Table S1

Table S2

Table S3

Table S4

Table S5

Table S6

## Data Availability

The data used to support the findings of this study are available from the corresponding author upon reasonable request.

## References

[1] Bowtell DD , Bohm S , Ahmed AA , et al. Rethinking ovarian cancer II: reducing mortality from high‐grade serous ovarian cancer. Nat Rev Cancer. 2015;15:668‐679. 26493647 10.1038/nrc4019 PMC4892184

[2] Lheureux S , Gourley C , Vergote I , Oza AM . Epithelial ovarian cancer. Lancet. 2019;393:1240‐1253. 30910306 10.1016/S0140-6736(18)32552-2

[3] Integrated genomic analyses of ovarian carcinoma. Nature. 2011;474:609‐615. 21720365 10.1038/nature10166 PMC3163504

[4] Patch AM , Christie EL , Etemadmoghadam D , et al. Whole‐genome characterization of chemoresistant ovarian cancer. Nature. 2015;521:489‐494. 26017449 10.1038/nature14410

[5] Levine AJ , Oren M . The first 30 years of p53: growing ever more complex. Nat Rev Cancer. 2009;9:749‐758. 19776744 10.1038/nrc2723 PMC2771725

[6] Levine AJ . The many faces of p53: something for everyone. J Mol Cell Biol. 2019;11:524‐530. 30925588 10.1093/jmcb/mjz026 PMC6736316

[7] Muller PA , Vousden KH . Mutant p53 in cancer: new functions and therapeutic opportunities. Cancer Cell. 2014;25:304‐317. 24651012 10.1016/j.ccr.2014.01.021 PMC3970583

[8] Zhou X , Hao Q , Lu H . Mutant p53 in cancer therapy‐the barrier or the path. J Mol Cell Biol. 2019;11:293‐305. 30508182 10.1093/jmcb/mjy072 PMC6487791

[9] Zhang Y , Cao L , Nguyen D , Lu H . TP53 mutations in epithelial ovarian cancer. Transl Cancer Res. 2016;5:650‐663. 30613473 10.21037/tcr.2016.08.40 PMC6320227

[10] Alsop K , Fereday S , Meldrum C , et al. BRCA mutation frequency and patterns of treatment response in BRCA mutation‐positive women with ovarian cancer: a report from the Australian Ovarian Cancer Study Group. J Clin Oncol. 2012;30:2654‐2663. 22711857 10.1200/JCO.2011.39.8545 PMC3413277

[11] Walsh T , Lee MK , Casadei S , et al. Detection of inherited mutations for breast and ovarian cancer using genomic capture and massively parallel sequencing. PNAS. 2010;107:12629‐12633. 20616022 10.1073/pnas.1007983107 PMC2906584

[12] Tothill RW , Tinker AV , George J , et al. Novel molecular subtypes of serous and endometrioid ovarian cancer linked to clinical outcome. Clin Cancer Res. 2008;14:5198‐5208. 18698038 10.1158/1078-0432.CCR-08-0196

[13] Izar B , Tirosh I , Stover EH , et al. A single‐cell landscape of high‐grade serous ovarian cancer. Nat Med. 2020;26:1271‐1279. 32572264 10.1038/s41591-020-0926-0 PMC7723336

[14] Geistlinger L , Oh S , Ramos M , et al. Multiomic analysis of subtype evolution and heterogeneity in high‐grade serous ovarian carcinoma. Cancer Res. 2020;80:4335‐4345. 32747365 10.1158/0008-5472.CAN-20-0521 PMC7572645

[15] Hu Z , Artibani M , Alsaadi A , et al. The repertoire of serous ovarian cancer non‐genetic heterogeneity revealed by single‐cell sequencing of normal fallopian tube epithelial cells. Cancer Cell. 2020;37:226‐242 e227. 32049047 10.1016/j.ccell.2020.01.003

[16] Hanahan D , Weinberg RA . Hallmarks of cancer: the next generation. Cell. 2011;144:646‐674. 21376230 10.1016/j.cell.2011.02.013

[17] Patel AP , Tirosh I , Trombetta JJ , et al. Single‐cell RNA‐seq highlights intratumoral heterogeneity in primary glioblastoma. Science. 2014;344:1396‐1401. 24925914 10.1126/science.1254257 PMC4123637

[18] Karnezis AN , Cho KR , Gilks CB , Pearce CL , Huntsman DG . The disparate origins of ovarian cancers: pathogenesis and prevention strategies. Nat Rev Cancer. 2017;17:65‐74. 27885265 10.1038/nrc.2016.113

[19] Ostrowski LE , Hutchins JR , Zakel K , O'Neal WK . Targeting expression of a transgene to the airway surface epithelium using a ciliated cell‐specific promoter. Mol Ther. 2003;8:637‐645. 14529837 10.1016/s1525-0016(03)00221-1

[20] Mikami Y , Iwase T , Komiyama Y , Matsumoto N , Oki H , Komiyama K . Secretory leukocyte protease inhibitor inhibits expression of polymeric immunoglobulin receptor via the NF‐kappaB signaling pathway. Mol Immunol. 2015;67:568‐574. 26239418 10.1016/j.molimm.2015.07.021

[21] Stuart T , Butler A , Hoffman P , et al. Comprehensive integration of single‐cell data. Cell. 2019;177:1888‐1902 e1821. 31178118 10.1016/j.cell.2019.05.031 PMC6687398

[22] van Hensbergen Y , Broxterman HJ , Rana S , et al. Reduced growth, increased vascular area, and reduced response to cisplatin in CD13‐overexpressing human ovarian cancer xenografts. Clin Cancer Res. 2004;10:1180‐1191. 14871998 10.1158/1078-0432.ccr-0482-3

[23] Ko SY , Guo H , Barengo N , Naora H . Inhibition of ovarian cancer growth by a tumor‐targeting peptide that binds eukaryotic translation initiation factor 4E. Clin Cancer Res. 2009;15:4336‐4347. 19458052 10.1158/1078-0432.CCR-08-2924

[24] Liu T , Wei Q , Jin J , et al. The m6A reader YTHDF1 promotes ovarian cancer progression via augmenting EIF3C translation. Nucleic Acids Res. 2020;48:3816‐3831. 31996915 10.1093/nar/gkaa048 PMC7144925

[25] Zhou X , Liao WJ , Liao JM , Liao P , Lu H . Ribosomal proteins: functions beyond the ribosome. J Mol Cell Biol. 2015;7:92‐104. 25735597 10.1093/jmcb/mjv014 PMC4481666

[26] Bywater MJ , Poortinga G , Sanij E , et al. Inhibition of RNA polymerase I as a therapeutic strategy to promote cancer‐specific activation of p53. Cancer Cell. 2012;22:51‐65. 22789538 10.1016/j.ccr.2012.05.019 PMC3749732

[27] Aibar S , Gonzalez‐Blas CB , Moerman T , et al. SCENIC: single‐cell regulatory network inference and clustering. Nat Methods. 2017;14:1083‐1086. 28991892 10.1038/nmeth.4463 PMC5937676

[28] Meng Q , Xia Y . c‐Jun, at the crossroad of the signaling network. Protein Cell. 2011;2:889‐898. 22180088 10.1007/s13238-011-1113-3 PMC4875184

[29] Aikawa Y , Morimoto K , Yamamoto T , et al. Treatment of arthritis with a selective inhibitor of c‐Fos/activator protein‐1. Nat Biotechnol. 2008;26:817‐823. 18587386 10.1038/nbt1412

[30] Khan I , Hossain A , Whitman GF , Sarkar NH , McDonough PG . Differential induction of c‐jun expression by PGF2‐alpha in rat ovary, uterus and adrenal. Prostaglandins. 1993;46:139‐144. 8210443 10.1016/0090-6980(93)90039-a

[31] Shelley ME , Hossain A , McDonough PG , Khan I . Differential c‐jun gene expression with tonically administered steroids in rat ovary and uterus. Am J Obstet Gynecol. 1994;170:1410‐1415. 8178882 10.1016/s0002-9378(94)70172-5

[32] Rusovici R , LaVoie HA . Expression and distribution of AP‐1 transcription factors in the porcine ovary. Biol Reprod. 2003;69:64‐74. 12606371 10.1095/biolreprod.102.013995

[33] Neyns B , Katesuwanasing VermeijJ , et al. Expression of the jun family of genes in human ovarian cancer and normal ovarian surface epithelium. Oncogene. 1996;12:1247‐1257. 8649827

[34] Chen X , Song E . Turning foes to friends: targeting cancer‐associated fibroblasts. Nat Rev Drug Discovery. 2019;18:99‐115. 30470818 10.1038/s41573-018-0004-1

[35] Efremova M , Vento‐Tormo M , Teichmann SA , Vento‐Tormo R . CellPhoneDB: inferring cell‐cell communication from combined expression of multi‐subunit ligand‐receptor complexes. Nat Protoc. 2020;15:1484‐1506. 32103204 10.1038/s41596-020-0292-x

[36] Gelmon KA , Tischkowitz M , Mackay H , et al. Olaparib in patients with recurrent high‐grade serous or poorly differentiated ovarian carcinoma or triple‐negative breast cancer: a phase 2, multicentre, open‐label, non‐randomised study. Lancet Oncol. 2011;12:852‐861. 21862407 10.1016/S1470-2045(11)70214-5

[37] Mateo J , Lord CJ , Serra V , et al. A decade of clinical development of PARP inhibitors in perspective. Ann Oncol. 2019;30:1437‐1447. 31218365 10.1093/annonc/mdz192 PMC6771225

[38] Puram SV , Tirosh I , Parikh AS , et al. Single‐cell transcriptomic analysis of primary and metastatic tumor ecosystems in head and neck cancer. Cell. 2017;171:1611‐1624 e1624. 29198524 10.1016/j.cell.2017.10.044 PMC5878932

[39] Ebright RY , Lee S , Wittner BS , et al. Deregulation of ribosomal protein expression and translation promotes breast cancer metastasis. Science. 2020;367:1468‐1473. 32029688 10.1126/science.aay0939 PMC7307008

[40] Fatica A , Tollervey D . Making ribosomes. Curr Opin Cell Biol. 2002;14:313‐318. 12067653 10.1016/s0955-0674(02)00336-8

[41] Dai MS , Zeng SX , Jin Y , Sun XX , David L , Lu H . Ribosomal protein L23 activates p53 by inhibiting MDM2 function in response to ribosomal perturbation but not to translation inhibition. Mol Cell Biol. 2004;24:7654‐7668. 15314173 10.1128/MCB.24.17.7654-7668.2004 PMC506971

[42] Dai MS , Lu H . Inhibition of MDM2‐mediated p53 ubiquitination and degradation by ribosomal protein L5. J Biol Chem. 2004;279:44475‐44482. 15308643 10.1074/jbc.M403722200

[43] Devlin JR , Hannan KM , Hein N , et al. Combination therapy targeting ribosome biogenesis and mRNA translation synergistically extends survival in MYC‐driven lymphoma. Cancer Discov. 2016;6:59‐70. 26490423 10.1158/2159-8290.CD-14-0673

[44] Zhang Y , Lu H . Signaling to p53: ribosomal proteins find their way. Cancer Cell. 2009;16:369‐377. 19878869 10.1016/j.ccr.2009.09.024 PMC4369769

[45] Zhou X , Hao Q , Zhang Q , et al. Ribosomal proteins L11 and L5 activate TAp73 by overcoming MDM2 inhibition. Cell Death Differ. 2015;22:755‐766. 25301064 10.1038/cdd.2014.167 PMC4392073

[46] Hao Q , Wang J , Chen Y , et al. Dual regulation of p53 by the ribosome maturation factor SBDS. Cell Death Dis. 2020;11:197. 32198344 10.1038/s41419-020-2393-4 PMC7083877

[47] Dai MS , Arnold H , Sun XX , Sears R , Lu H . Inhibition of c‐Myc activity by ribosomal protein L11. EMBO J. 2007;26:3332‐3345. 17599065 10.1038/sj.emboj.7601776 PMC1933407

[48] Zhou X , Hao Q , Liao JM , Liao P , Lu H . Ribosomal protein S14 negatively regulates c‐Myc activity. J Biol Chem. 2013;288:21793‐21801. 23775087 10.1074/jbc.M112.445122 PMC3724636

[49] Papavassiliou AG , Musti AM . The multifaceted output of c‐jun biological activity: focus at the junction of CD8 T cell activation and exhaustion. Cells. 2020;9. 10.3390/cells9112470 PMC7697663 33202877

[50] Beenken A , Mohammadi M . The FGF family: biology, pathophysiology and therapy. Nat Rev Drug Discovery. 2009;8:235‐253. 19247306 10.1038/nrd2792 PMC3684054

[51] Papadopoulos N , Lennartsson J . The PDGF/PDGFR pathway as a drug target. Mol Aspects Med. 2018;62:75‐88. 29137923 10.1016/j.mam.2017.11.007

[52] Lee KM , Danuser R , Stein JV , Graham D , Nibbs RJ , Graham GJ . The chemokine receptors ACKR2 and CCR2 reciprocally regulate lymphatic vessel density. EMBO J. 2014;33:2564‐2580. 25271254 10.15252/embj.201488887 PMC4283412

[53] Hansell CAH , Fraser AR , Hayes AJ , et al. The atypical chemokine receptor Ackr2 constrains NK cell migratory activity and promotes metastasis. J Immunol. 2018;201:2510‐2519. 30158126 10.4049/jimmunol.1800131 PMC6176105

[54] Butler A , Hoffman P , Smibert P , Papalexi E , Satija R . Integrating single‐cell transcriptomic data across different conditions, technologies, and species. Nat Biotechnol. 2018;36:411‐420. 29608179 10.1038/nbt.4096 PMC6700744

[55] Macosko EZ , Basu A , Satija R , et al. Highly parallel genome‐wide expression profiling of individual cells using nanoliter droplets. Cell. 2015;161:1202‐1214. 26000488 10.1016/j.cell.2015.05.002 PMC4481139

[56] Haghverdi L , Lun ATL , Morgan MD , Marioni JC . Batch effects in single‐cell RNA‐sequencing data are corrected by matching mutual nearest neighbors. Nat Biotechnol. 2018;36:421‐427. 29608177 10.1038/nbt.4091 PMC6152897

[57] Trapnell C , Cacchiarelli D , Grimsby J , et al. The dynamics and regulators of cell fate decisions are revealed by pseudotemporal ordering of single cells. Nat Biotechnol. 2014;32:381‐386. 24658644 10.1038/nbt.2859 PMC4122333

[58] Yang Y , Guo F , Peng Y , et al. Transcriptomic profiling of human placenta in gestational diabetes mellitus at the single‐cell level. Front Endocrinol. 2021;12:679582. 10.3389/fendo.2021.679582 PMC8139321 34025588

[59] Chen Y , Hao Q , Wang J , et al. Ubiquitin ligase TRIM71 suppresses ovarian tumorigenesis by degrading mutant p53. Cell Death Dis. 2019;10:737. 31570706 10.1038/s41419-019-1977-3 PMC6769007

[60] Huang C , Hao Q , Shi G , Zhou X , Zhang Y . BCL7C suppresses ovarian cancer growth by inactivating mutant p53. J Mol Cell Biol. 2021;13:141‐150. 33306126 10.1093/jmcb/mjaa065 PMC8104935

[61] Gyorffy B , Lanczky A , Szallasi Z . Implementing an online tool for genome‐wide validation of survival‐associated biomarkers in ovarian‐cancer using microarray data from 1287 patients. Endocr Relat Cancer. 2012;19:197‐208. 22277193 10.1530/ERC-11-0329

