# Supplementary material for: Single‐cell transcriptomes reveal heterogeneity of high‐grade serous ovarian carcinoma
Source: Clin Transl Med. 2021 Aug 4;11(8):e500. doi: 10.1002/ctm2.500 (PMC8335963; doi:10.1002/ctm2.500)

Supplementary Information

Single-cell transcriptomes reveal heterogeneity of high-grade serous ovarian carcinoma

Qian Hao[1,3,#], Jiajia Li[2,3,#], Qinghua Zhang[1,3,#], Fei Xu[2,3,#], Bangxiang Xie[4], Hua Lu[5], Xiaohua Wu[2,3,*], and Xiang Zhou[1,3,6,7,*]

[1] Fudan University Shanghai Cancer Center and Institutes of Biomedical Sciences, Fudan University, Shanghai 200032, China

[2] Department of Gynecologic Oncology, Fudan University Shanghai Cancer Center, Fudan University, Shanghai 200032, China

[3] Department of Oncology, Shanghai Medical College, Fudan University, Shanghai 200032, China

[4] Beijing YouAn Hospital, Capital Medical University, Beijing Institute of Hepatology, Beijing 100069, China

[5] Department of Biochemistry & Molecular Biology and Tulane Cancer Center, Tulane University School of Medicine, New Orleans, LA 70112, USA

[6] Key Laboratory of Breast Cancer in Shanghai, Fudan University Shanghai Cancer Center, Fudan University, Shanghai, 200032, China

[7] Shanghai Key Laboratory of Medical Epigenetics, International Co-laboratory of Medical Epigenetics and Metabolism, Ministry of Science and Technology, Institutes of Biomedical Sciences, Fudan University, Shanghai 200032, China

[#] Equal contribution

[*] Correspondence:

Xiaohua Wu, Department of Gynecologic Oncology, Fudan University Shanghai Cancer Center, Fudan University, Shanghai 200032, China. Email: wu.xh@fudan.edu.cn

Xiang Zhou, Fudan University Shanghai Cancer Center and Institutes of Biomedical Sciences, Fudan University, Shanghai 200032, China. Email: xiangzhou@fudan.edu.cn

Running title: Single-cell RNA-seq of HGSOC

Supplementary figure legends

Figure S1. (A) Percentile distribution of the nine cell clusters in primary (POC1 and POC2) and metastatic (MOC1 and MOC2) ovarian carcinomas. (B) The t-SNE plot shows distribution of all the single cells from four samples. (C) The t-SNE plot shows distribution of all the single cells from primary and metastatic foci. (D-I) Violin plots show the expression of selected markers in each cell type. (J) The t-SNE plot shows CNV levels of different cell clusters. (K) Violin plots show CNV levels among six cell types in primary and metastatic tumors, respectively.

Figure S2. (A) Percentile distribution of the five subclusters of epithelial cells in primary (POC1 and POC2) and metastatic (MOC1 and MOC2) ovarian carcinomas. (B) The t-SNE plot shows distribution of epithelial cells from four samples. (C) The t-SNE plot shows distribution of primary and metastatic epithelial cells. (D) The t-SNE plot shows CNV levels of epithelial cell subclusters. (E) Violin plots show CNV levels among five epithelial cell subclusters in primary and metastatic tumors, respectively. (F-H) Violin plots show the expression of selected markers in each subset of epithelial cells. (I, J) IHC staining of MUC5B, FOXD1, PAEP, and HP in the primary and metastatic tumor samples. (K) Violin plots show the expression of selected markers for functional validation shown in Figures 3 and S3.

Figure S3. (A) Ablation of any of the newly identified marker genes moderately regulates growth of colorectal cancer RKO cells. (B) Ablation of any of the newly identified marker genes moderately regulates growth of breast cancer MCF-7 cells. *$p<0.05$ by two tailed t-test.

Figure S4. (A) Percentile distribution of the five subclusters of fibroblast cells in primary (POC1 and POC2) and metastatic (MOC1 and MOC2) ovarian carcinomas. (B) The t-SNE plot shows distribution of fibroblast cells from four samples. (C) The t-SNE plot shows distribution of fibroblast cells from primary and metastatic foci. (D) The heatmap shows the correlation of subclusters between epithelial cells and fibroblast cells. Color key from white to blue indicates correlation levels from low to high. (E) Violin plots show the expression of selected epithelial markers and EMT markers. (F, G) IHC staining of STAR and MFAP5 in the primary and metastatic tumor samples.

Figure S5. The JUN/AP-1 inhibitor T-5224 suppresses proliferation of normal ovarian epithelial IOSE cells. *$p<0.05$ by two tailed t-test.

Figure S1

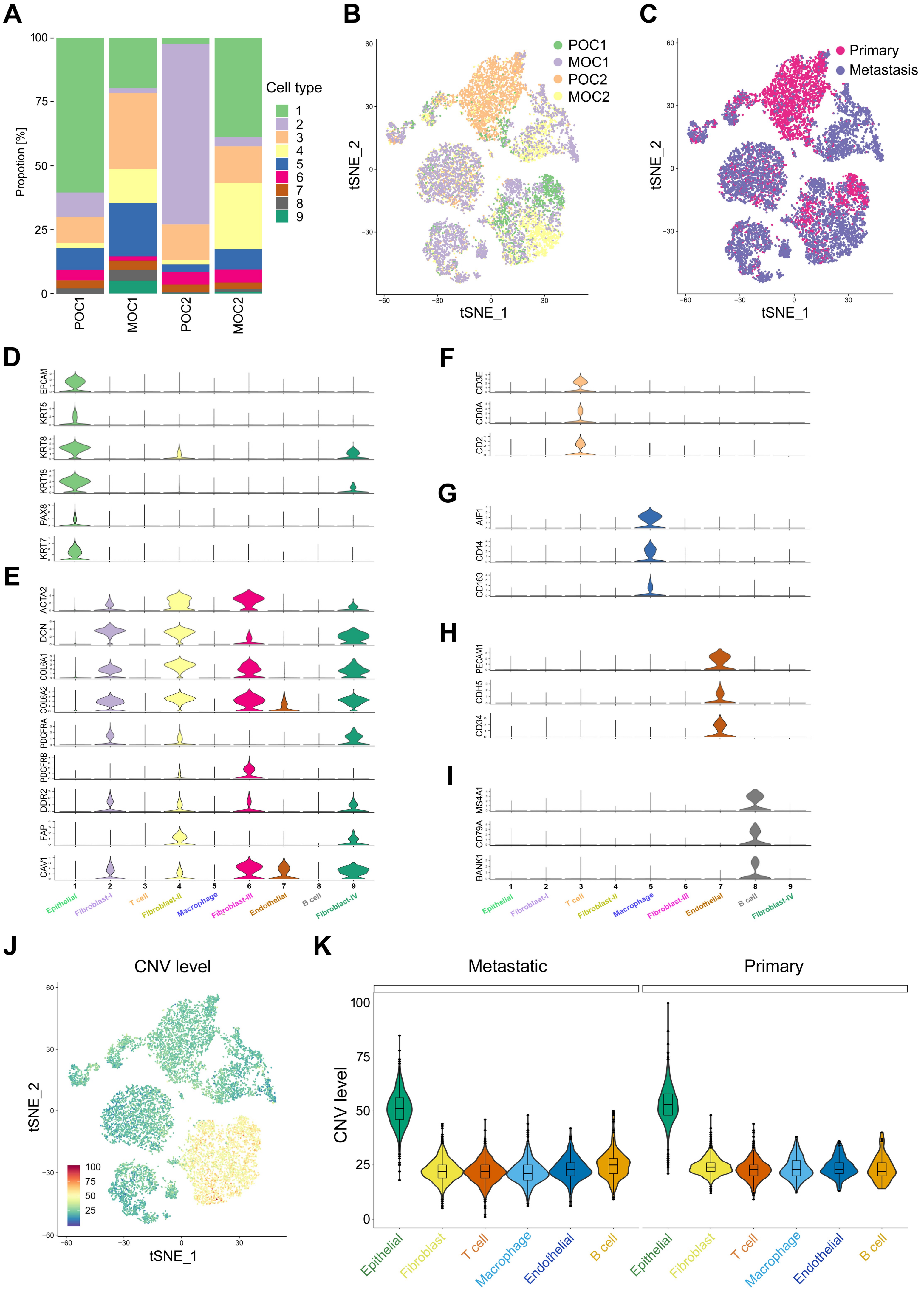

A
B
C
D
E
F
G
H
I
J
K
Propotion [%]
Cell type
POC1
MOC1
POC2
MOC2
Primary
Metastasis
tSNE_1
tSNE_2
EPCAM
KRT5
KRT8
KRT18
PAX8
KRT7
ACTA2
DCN
COL6A1
COL6A2
PDGFRA
PDGFRB
DDR2
FAP
CAV1
CD3E
CD8A
CD2
AIF1
CD14
CD163
PECAM1
CDH5
CD34
MS4A1
CD79A
BANK1
Epithelial
Fibroblast-I
T cell
Fibroblast-II
Macrophage
Fibroblast-III
Endothelial
B cell
Fibroblast-IV
CNV level
Metastatic
Primary
Epithelial
Fibroblast
T cell
Macrophage
Endothelial
B cell

Figure S2

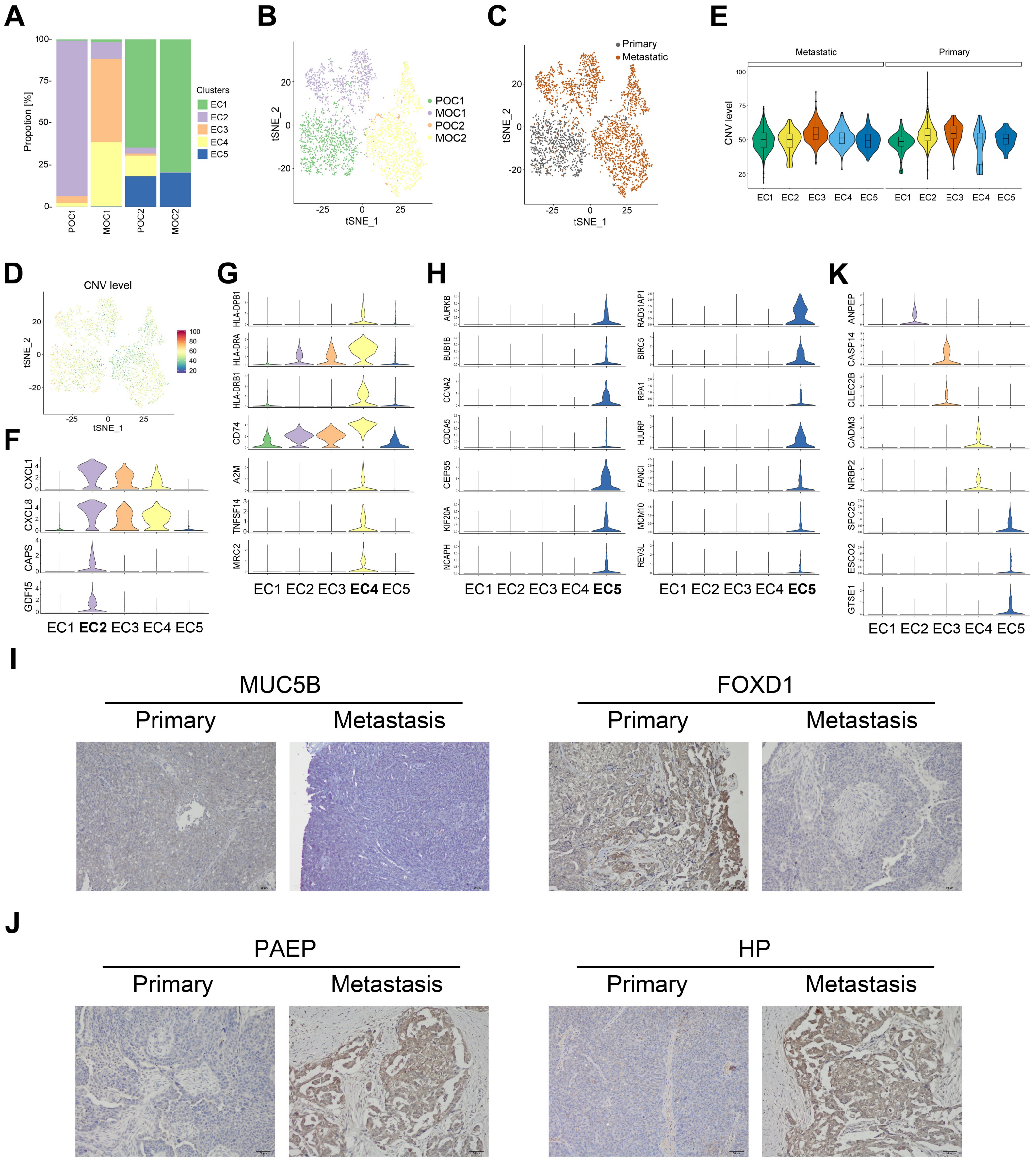

A
B
C
E
D
F
G
H
K
I
J
Clusters
EC1
EC2
EC3
EC4
EC5
Propotion [%]
POC1
MOC1
POC2
MOC2
tSNE_1
tSNE_2
Primary
Metastatic
CNV level
CXCL1
CXCL8
CAPS
GDF15
EC1 EC2 EC3 EC4 EC5
HLA-DPB1
HLA-DRA
HLA-DRB1
CD74
A2M
TNFSF14
MRC2
AURKB
BUB1B
CCNA2
CDCA5
CEP55
KIF20A
NCAPH
RAD51AP1
BIRC5
RPA1
HJURP
FANCI
MCM10
REV3L
ANPEP
CASP14
CLEC2B
CADM3
NRBP2
SPC25
ESCO2
GTSE1
MUC5B
FOXD1
PAEP
HP
Primary
Metastasis

Figure S3

A

RKO cells

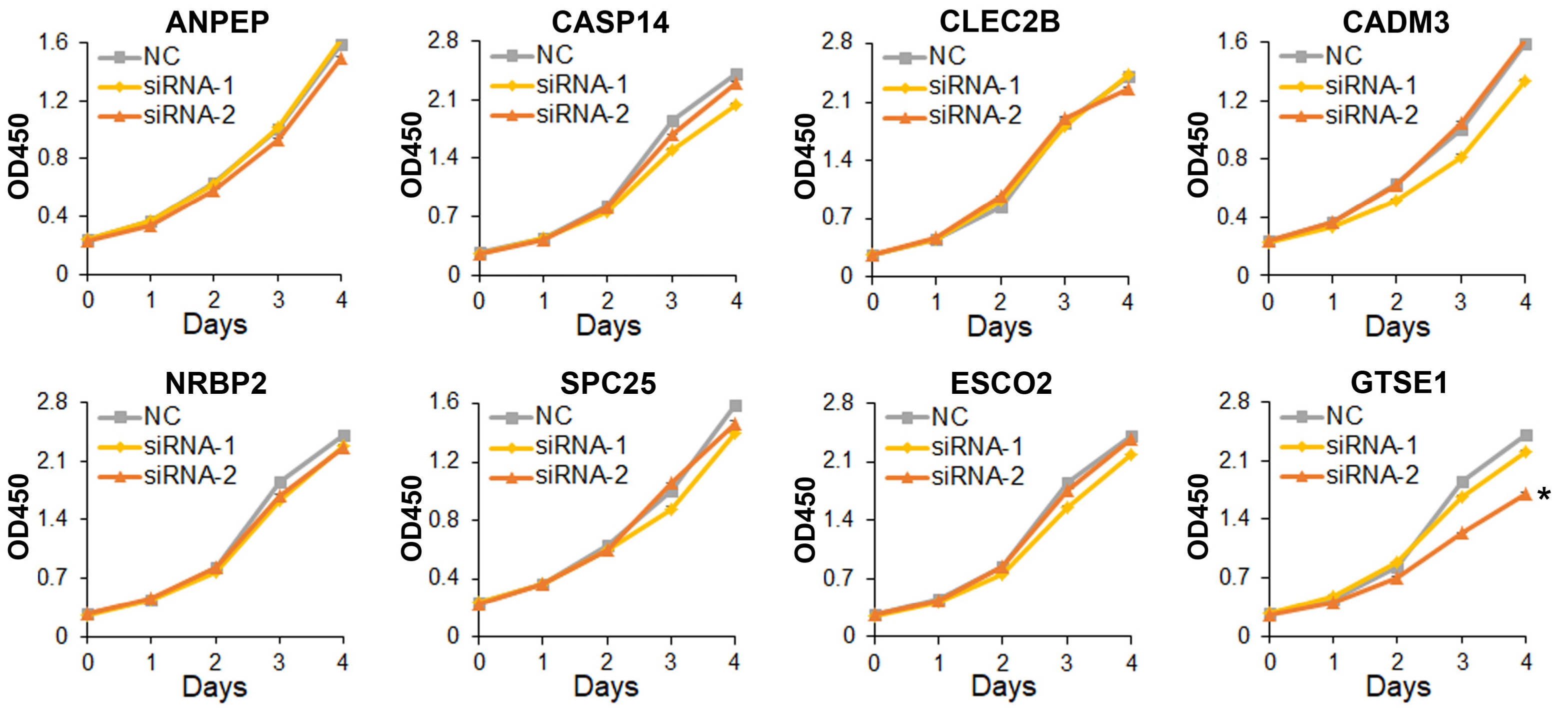

B

MCF-7 cells

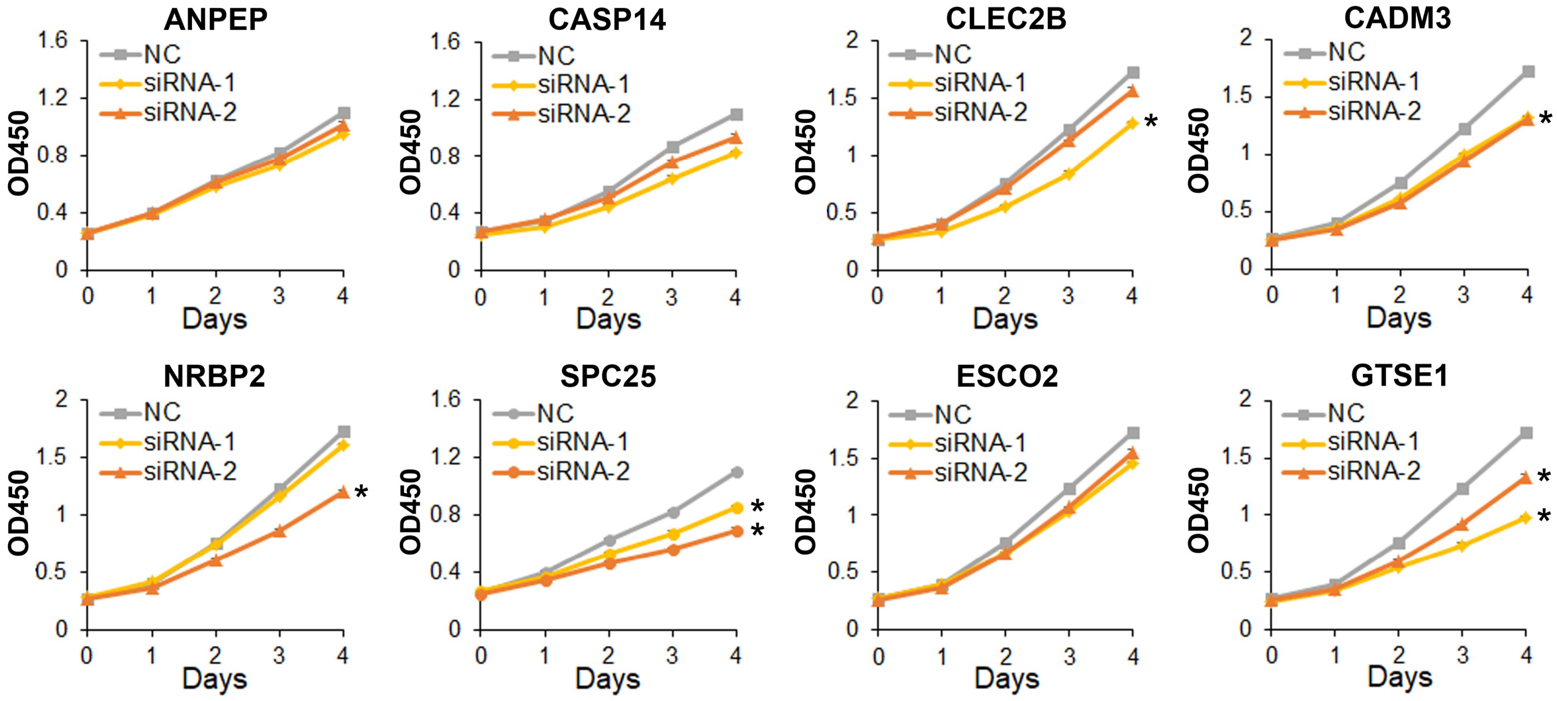

Figure S4

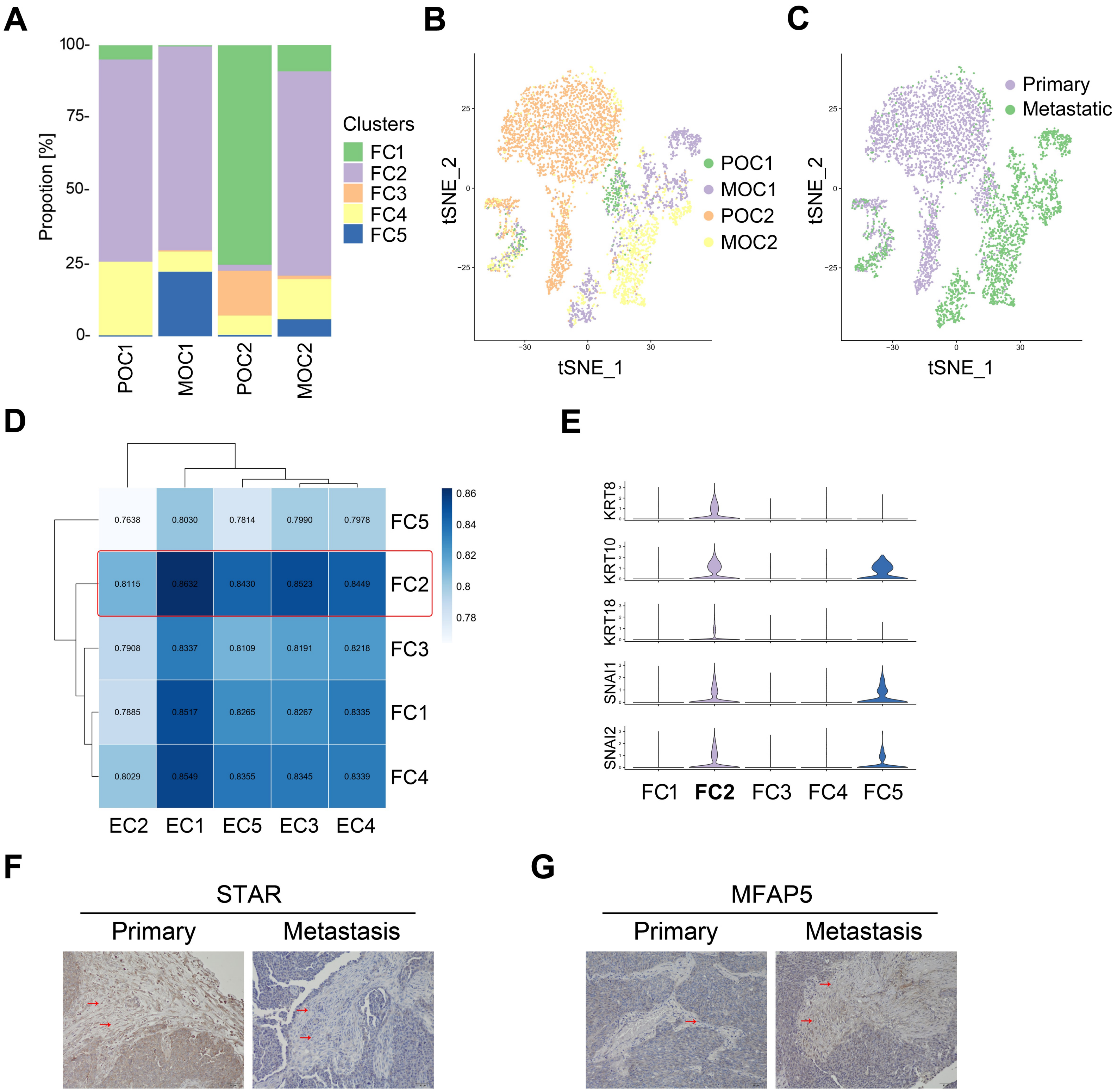
A
B
C
Propotion [%]
100-
75-
50-
25-
0-
POC1
MOC1
POC2
MOC2
Clusters
FC1
FC2
FC3
FC4
FC5
tSNE_2
tSNE_1
POC1
MOC1
POC2
MOC2
Primary
Metastatic
D
E
0.7638 0.8030 0.7814 0.7990 0.7978 FC5
0.8115 0.8632 0.8430 0.8523 0.8449 FC2
0.7908 0.8337 0.8109 0.8191 0.8218 FC3
0.7885 0.8517 0.8265 0.8267 0.8335 FC1
0.8029 0.8549 0.8355 0.8345 0.8339 FC4
EC2 EC1 EC5 EC3 EC4
0.86
0.84
0.82
0.8
0.78
KRT8
KRT10
KRT18
SNAI1
SNAI2
FC1 FC2 FC3 FC4 FC5
F
STAR
Primary
Metastasis
G
MFAP5
Primary
Metastasis

Figure S5

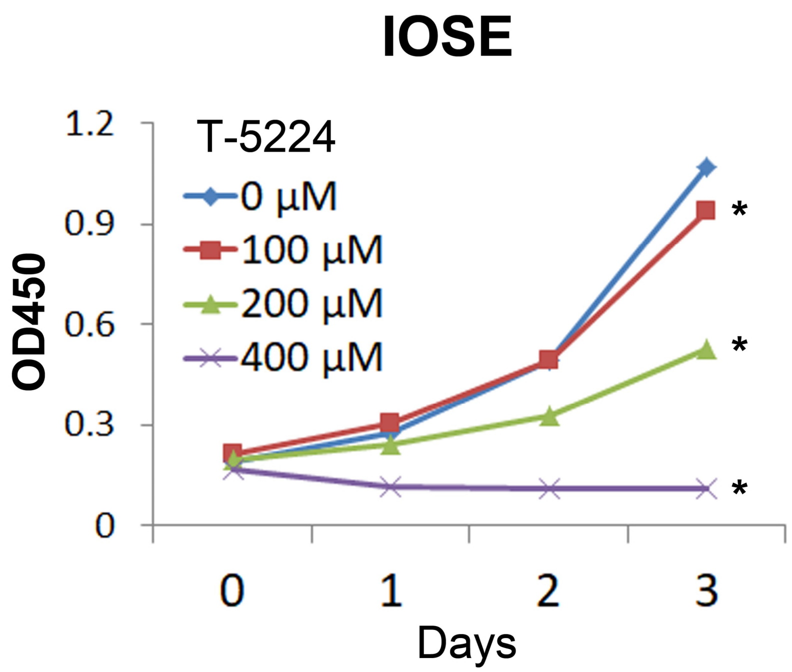

Supplement: Supplementary file 1 — Supporting Information [file CTM2-11-e500-s007.pdf]
